# TLC-Derived High-Polar Fractions of *Celastrus paniculatus* Seeds Attenuate Astrocyte-Driven Microglial Activation Through Suppression of CD40/iNOS Signaling and Pro-Inflammatory Cytokines

**DOI:** 10.3390/ijms27083551

**Published:** 2026-04-16

**Authors:** Tanida Treerattanakulporn, Narongrit Thongon, Siriporn Chamniansawat

**Affiliations:** Department of Medical Sciences, Faculty of Allied Health Sciences, Burapha University, Chonburi 20131, Thailand; tanidatrirattanakulporn@gmail.com (T.T.); narongritt@buu.ac.th (N.T.)

**Keywords:** *Celastrus paniculatus*, microglial activation, astrocyte-conditioned medium, neuroinflammation, CD40 signaling, inducible nitric oxide synthase (iNOS), LC–MS/MS metabolomics

## Abstract

Neuroinflammation mediated by astrocyte–microglia interactions plays a critical role in the progression of neurodegenerative disorders. *Celastrus paniculatus* (CP) seeds have long been associated with cognitive benefits; however, the chemical composition and anti-inflammatory potential of their high-polarity fractions remain poorly characterized. In this study, thin-layer chromatography (TLC)-derived high-polarity fractions (F6 and F7) from CP seeds were analyzed using untargeted LC–MS/MS metabolite profiling. After quality filtering, 99 metabolites were retained for classification, with enrichment of alkaloids and terpenoid-related compounds, including 41 structurally complex metabolites. To evaluate biological relevance, BV2 microglia were exposed to astrocyte-conditioned medium derived from H_2_O_2_-treated astrocytes (ACM-H), modeling sterile inflammatory signaling. ACM-H stimulation induced microglial activation characterized by morphological transformation, increased CD40 and inducible nitric oxide synthase (iNOS) expression, and elevated production of pro-inflammatory cytokines TNF-α and IL-6. Co-treatment with CP fractions attenuated ACM-H-induced inflammatory responses, with fraction F7 showing stronger effects than F6. Fraction F7 showed stronger inhibitory effects on CD40 and iNOS expression, suppressed TNF-α and IL-6 production, and partially restored ramified microglial morphology, whereas F6 exhibited comparable anti-inflammatory activity and showed a stronger effect on microglial phagocytic responses. Metabolomic analysis further indicated a higher relative abundance of terpenoid-related metabolites in F7. Collectively, these findings indicate that CP seed fractions, particularly F7, attenuate astrocyte-driven microglial activation in an in vitro sterile neuroinflammatory model.

## 1. Introduction

Microglia regulate CNS inflammation and transition from a surveillant to an activated phenotype in response to injury or stress, accompanied by changes in cytokine secretion and phagocytic function [1,2,3]. This phenotypic transition is initiated by pattern-recognition mechanisms that detect endogenous stress signals and activate intracellular inflammatory pathways, leading to sustained production of pro-inflammatory mediators [3]. Although lipopolysaccharide (LPS) is widely used to induce pro-inflammatory activation in vitro, it represents a pathogen-associated molecular pattern (PAMP)-driven stimulus that may not fully recapitulate sterile inflammatory conditions characteristic of neurodegeneration [4,5]. In contrast, astrocyte-conditioned medium derived from H_2_O_2_-treated astrocytes (ACM-H) provides a physiologically relevant model of glial communication, as stressed astrocytes release cytokines, chemokines, reactive oxygen species, and endogenous signals that modulate microglial phenotype [6]. These responses resemble sterile inflammatory signaling in neurodegenerative conditions, where damage-associated molecular pattern (DAMP) release and downstream signaling perpetuate microglial activation in the absence of microbial infection [5]. Therefore, ACM-based stimulation offers an advantage for modeling sterile neuroinflammatory signaling rather than direct endotoxin-induced activation.

Upon inflammatory stimulation, microglia upregulate inflammatory mediators and activation markers, including CD40, inducible nitric oxide synthase (iNOS), and pro-inflammatory cytokines such as TNF-α and IL-6 [2,7,8]. This phenotype is accompanied by morphological remodeling, including enlarged cell bodies, retracted processes, and reduced ramification, reflecting a shift from a ramified morphology to an amoeboid or phagocytic state of activated microglia [9]. These alterations reflect enhanced inflammatory signaling and phagocytic activity, processes that may contribute to neuronal injury during neuroinflammation [10]. Collectively, these observations highlight the importance of modulating astrocyte-driven microglial activation, particularly CD40 expression, inflammatory cytokine release, and phagocytic remodeling under sterile neuroinflammatory conditions.

*Celastrus paniculatus* (CP), a medicinal plant belonging to the Celastraceae family, has long been used in traditional medicine for neurological and inflammatory disorders [11]. Phytochemical investigations indicate that CP seeds contain structurally diverse bioactive constituents, including sesquiterpene alkaloids, sesquiterpene polyol esters, triterpenoids, sterols, and related terpenoid derivatives [12]. However, despite numerous reports describing the overall phytochemical profile of CP seeds, the characterization of biologically active, polarity-defined fractions and their relevance to astrocyte-driven microglial activation under sterile neuroinflammatory conditions remains poorly defined. In our previous study, solvent-partitioned fractions of CP seed extract exhibited polarity-dependent biological activities [13], consistent with earlier reports demonstrating the neuroprotective and antioxidant effects of CP extracts in neuronal models and experimental animals [14,15]. Notably, ethyl acetate and aqueous fractions showed enhanced neuroprotective and antioxidant properties compared with crude extract and non-polar fractions. These findings suggest that polarity-defined fractions of CP may concentrate bioactive constituents responsible for neuroprotective effects, providing a rationale for further chemical and functional characterization of high-polar fractions.

Therefore, the present study aimed to investigate the immunomodulatory effects of high-polar fractions of CP seeds in a physiologically relevant astrocyte-driven neuroinflammatory model. We further characterized microglial activation phenotypes, including morphological remodeling, CD40/iNOS signaling, pro-inflammatory cytokine release, and phagocytic activity. In parallel, untargeted LC–MS/MS metabolite profiling was performed to identify putative bioactive constituents in fractions F6 and F7.

## 2. Results

### 2.1. LC-MS/MS-Based Metabolite Profiling of CP Seed Fractions

LC–MS/MS-based metabolite profiling was employed to characterize the chemical composition of the TLC-derived F6 and F7 fractions from CP seed extract. The two fractions appeared largely comparable, as indicated by similar total ion chromatograms and overall ion abundance patterns (Supplementary Figure S1), suggesting broadly overlapping metabolite profiles. In total, 737 metabolite features were detected in positive ion mode and 499 in negative ion mode. For downstream annotation and classification, only positive-mode features were retained because this mode provided higher sensitivity and more reliable MS/MS spectral matching for secondary metabolites.

**Figure 1:**
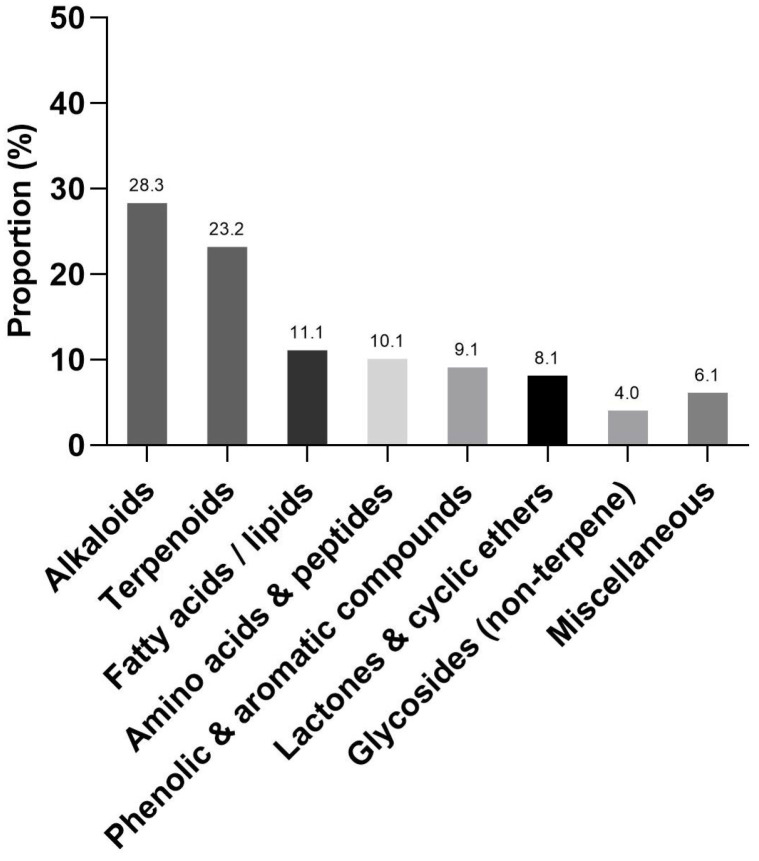
Chemical composition of fractions F6 and F7 derived from *Celastrus paniculatus* seeds. The bar graph shows the relative proportion (%) of metabolites classified into chemical classes based on untargeted LC–MS/MS analysis of fractions F6 and F7. A total of 99 metabolites that met predefined quality-filtering criteria were included in the classification. Metabolites were categorized into alkaloids, terpenoids/terpene-related compounds, fatty acids/lipids, amino acids & peptides, phenolic & aromatic compounds, lactones & cyclic ethers, glycosides (non-terpene), and miscellaneous compounds. Percentages indicate the relative distribution of metabolite counts within each chemical class.

To improve analytical robustness, a predefined quality-filtering pipeline was applied to the positive-mode dataset (see Methods), yielding 99 putatively annotated metabolites for subsequent chemical classification (Table S1). The filtered metabolites were categorized into major chemical classes based on structural ontology. Using the total number of filtered metabolites as 100%, alkaloids (28.3%) and terpenoid-related compounds (23.2%) represented the predominant classes, followed by fatty acids and lipid-related metabolites (11.1%), amino acids/peptides/nucleobase-related compounds (10.1%), phenolic and aromatic compounds (9.1%), lactones and cyclic ethers (8.1%), non-terpene glycosides (4.0%), and miscellaneous compounds (6.1%) (Figure 1). Overall, alkaloids and terpenoid-related metabolites constituted the largest proportion of structurally complex secondary metabolites within the bioactive fractions.

**Figure 2:**
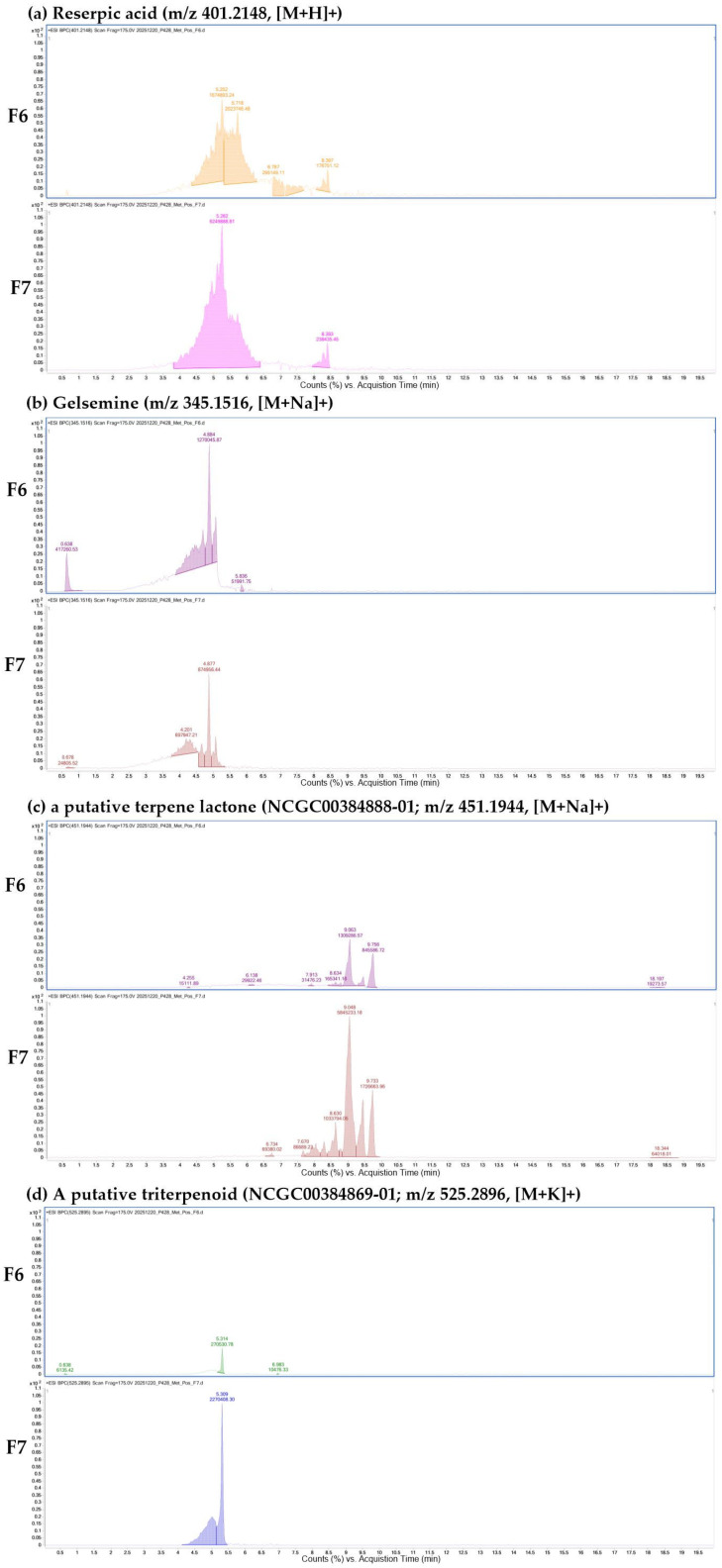
Representative extracted ion chromatograms (XICs) of selected metabolites detected in fractions F6 and F7 of CP seed extracts by LC–MS/MS. (**a**) Putative reserpic acid (corynanthean-type alkaloid; *m*/*z* 401.2148, [M+H]^+^), (**b**) putative gelsemine (gelsemium alkaloid; *m*/*z* 345.1516, [M+Na]^+^), (**c**) putative terpene lactone (NCGC00384888-01; *m*/*z* 451.1944, [M+Na]^+^), and (**d**) putative triterpenoid (NCGC00384869-01; *m*/*z* 525.2896, [M+K]^+^). Extracted ion chromatograms from fractions F6 and F7 are shown for comparison.

The refined dataset revealed structurally diverse secondary metabolites belonging to several major chemical classes, including multiple alkaloid subclasses, terpenoid-related compounds across different structural lineages, and phenolic glycosides and flavonoids (Table 1). Alkaloids included putatively annotated representatives from several structural families such as corynanthean-type, protoberberine, aporphine, and indole alkaloids, whereas terpenoid-related metabolites encompassed terpene lactones, diterpenoids, triterpenoids, sesquiterpene derivatives, and terpene glycosides. In addition, phenylpropanoid glycosides and prenylated flavonoids were tentatively assigned among the phenolic constituents. Together, these results indicate the chemical diversity of the high-polar fractions and provide a compositional context for interpreting the observed biological responses in microglial activation. Although overall metabolite classes were comparable, differences in the relative abundance of selected metabolites were observed between F6 and F7, as illustrated by representative XIC profiles (Figure 2).

### 2.2. Representative Extracted Ion Chromatograms of Major Metabolites in F6 and F7 Fractions

To illustrate the presence of selected metabolites, representative extracted ion chromatograms (XICs) were generated for alkaloid- and terpenoid-related features detected in the positive ion mode dataset (Figure 2). As shown in Figure 2a, the XIC corresponding to reserpic acid (*m*/*z* 401.2148, [M+H]^+^), a corynanthean-type alkaloid, was detected in both F6 and F7 fractions. Similarly, gelsemine (*m*/*z* 345.1516, [M+Na]^+^), a gelsemium-type alkaloid, was observed in both fractions (Figure 2b), indicating the presence of indole alkaloid-related features. In addition to alkaloids, terpenoid-related features were also detected in both fractions. A putative triterpenoid (NCGC00384869-01; *m*/*z* 525.2896, [M+K]^+^) and a putative terpene lactone glycoside (NCGC00384888-01; *m*/*z* 451.1944, [M+Na]^+^) showed detectable chromatographic peaks in both F6 and F7 fractions (Figure 2c,d). These XIC profiles support the presence of representative alkaloid- and terpenoid-related features in the analyzed fractions. However, XIC-based observations are not intended for quantitative comparison and should be interpreted as qualitative evidence of metabolite detection.

### 2.3. Dose–Response Effects of F6 and F7 on BV-2 Cell Viability

**Figure 3:**
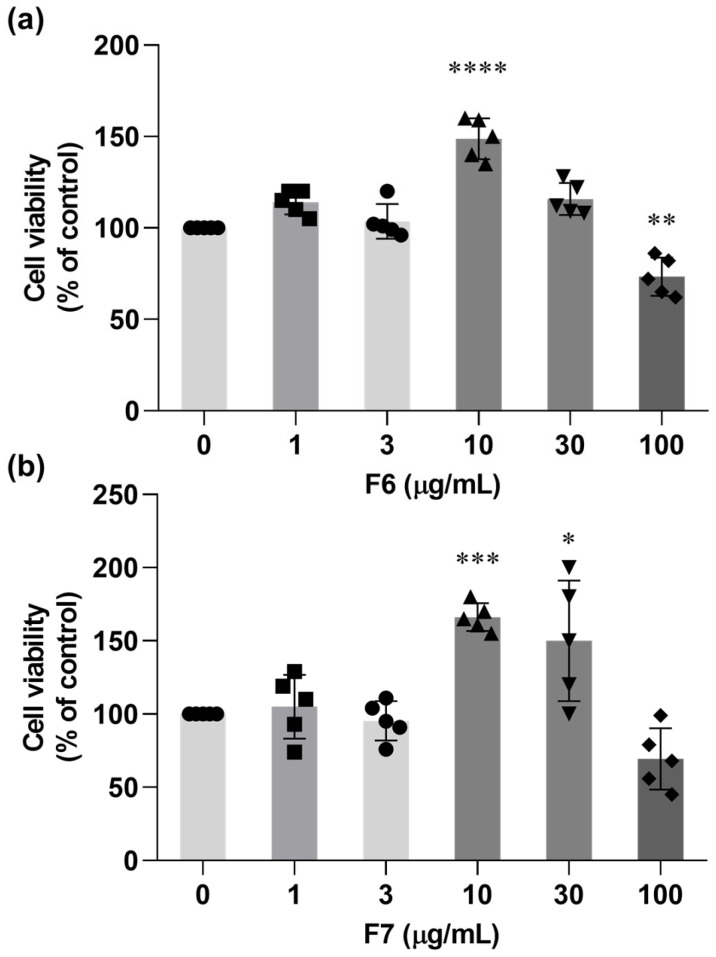
Effects of F6 and F7 on BV-2 cell viability. BV-2 cells were treated with increasing concentrations (1–100 µg/mL) of (**a**) F6 and (**b**) F7 for 24 h, and cell viability was assessed using the MTT assay. Data are expressed as percentage of control and presented as mean ± SEM from five independent biological experiments (n = 5). * *p* < 0.05, ** *p* < 0.01, *** *p* < 0.001, **** *p* < 0.0001 vs. control.

To determine the appropriate working concentration and assess potential cytotoxicity, BV-2 cells were treated with increasing concentrations of F6 and F7 (1–100 µg/mL), and cell viability was evaluated. As shown in Figure 3a,b, both F6 and F7 exhibited concentration-dependent effects on BV-2 cell viability. Treatment with low to moderate concentrations (1–30 µg/mL) did not reduce cell viability, and in some cases slightly increased viability compared to the control. Notably, the highest viability was observed at 10 µg/mL for both fractions. In contrast, treatment with 100 µg/mL resulted in a marked reduction in cell viability, indicating potential cytotoxic effects at higher concentrations. Based on these findings, 10 µg/mL was selected for subsequent experiments.

### 2.4. Establishment of Astrocyte-Conditioned Medium (ACM-H)

To generate a model of astrocyte-mediated sterile inflammatory signaling, CTX TNA2 astrocytes were transiently exposed to H_2_O_2_ followed by a recovery period, and the conditioned medium (ACM-H) was collected. Astrocytes subjected to this treatment exhibited features consistent with a stress-induced reactive phenotype, including altered mitochondrial function and increased expression of activation markers (Supplementary Figure S2), supporting the use of ACM-H in subsequent experiments.

### 2.5. CP Fractions Modulate ACM-H-Induced Microglial Morphological Activation and Associated Quantitative Parameters

**Figure 4:**
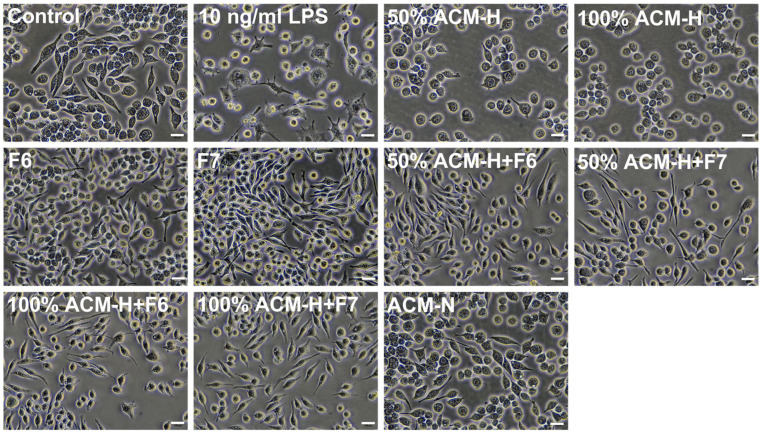
CP fractions attenuate ACM-H-induced microglial morphological alterations. Representative phase-contrast images showing microglial morphology under control conditions, following stimulation with LPS (10 ng/mL), ACM-H (50% and 100%), and treatment with CP seed fractions F6 or F7 alone. Co-treatment groups include ACM-H (50% and 100%) combined with F6 or F7. ACM-N was included as a non-stressed astrocyte-conditioned control. Scale bar = 100 µm.

Phase-contrast imaging revealed distinct morphological changes in microglia under different experimental conditions (Figure 4). Under control conditions, microglia displayed a ramified morphology with elongated processes. Stimulation with LPS (10 ng/mL) induced an amoeboid phenotype characterized by enlarged cell bodies and reduced processes. Exposure to ACM-H at both 50% and 100% concentrations produced marked morphological changes, including increased cell body size and reduced process ramification compared with control cells. Co-treatment with CP fractions F6 or F7 markedly attenuated ACM-H-induced morphological activation. Microglia treated with 50% or 100% ACM-H in the presence of F6 or F7 exhibited reduced cell body enlargement and adopted a more elongated, ramified morphology resembling that of resting microglia. In contrast, ACM derived from normal astrocytes (ACM-N) did not alter microglial morphology.

**Figure 5:**
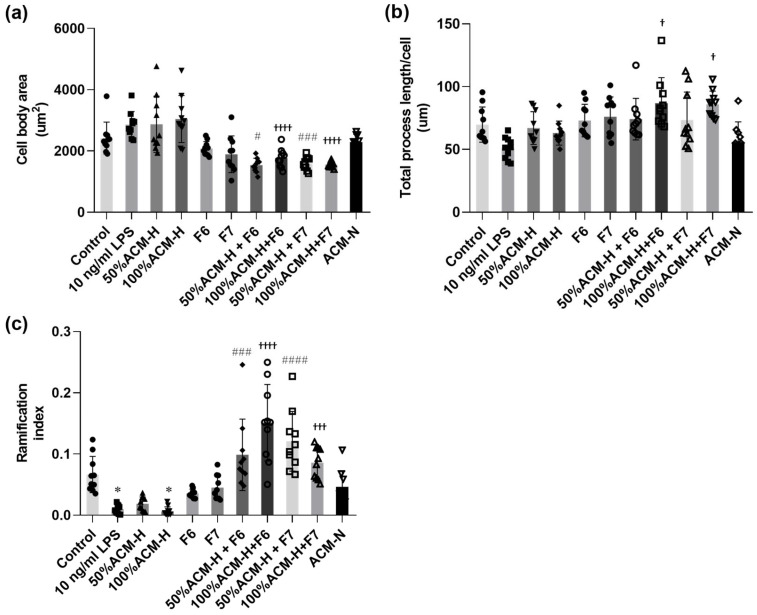
Quantitative analysis of the modulatory effects of CP fractions on ACM-H-induced microglial morphological alterations. Quantitative morphometric analysis of microglial morphology under control conditions, after stimulation with LPS, ACM-H (50% and 100%), treatment with CP seed fractions F6 or F7 alone, ACM-H co-treated with CP seed fractions F6 or F7, and ACM-N. Morphological parameters analyzed include (**a**) cell body area, (**b**) total process length per cell, and (**c**) ramification index. Morphometric measurements were obtained from phase-contrast images and quantified using using ImageJ software (version 1.53). All analyses were performed in a blinded manner. Data are presented as mean ± SEM from independent biological replicates (n = 10 cells per condition per experiment). * *p* < 0.05, vs. control; ^#^ *p* < 0.05, ^###^ *p* < 0.001, ^####^ *p* < 0.0001 vs. 50% ACM-H; ^†^ *p* < 0.05, ^†††^ *p* < 0.001, ^††††^ *p* < 0.0001 vs. 100% ACM-H.

Quantitative morphometric analysis supported the morphological findings (Figure 5). ACM-H significantly increased microglial cell body area (Figure 5a) and reduced total process length (Figure 5b) and ramification index (Figure 5c) compared with control cells. In contrast, treatment with CP fractions F6 or F7 alone did not markedly alter microglial morphology under basal conditions, as reflected by comparable cell body area, process length, and ramification index relative to control cells. Co-treatment with F6 or F7 attenuated ACM-H-induced morphological alterations, as evidenced by reduced cell body area and increased total process length and ramification index compared with ACM-H–treated cells.

### 2.6. CP Fractions Suppress ACM-H-Induced Pro-Inflammatory Activation of Microglia via Regulation of CD40 and iNOS Expression

**Figure 6:**
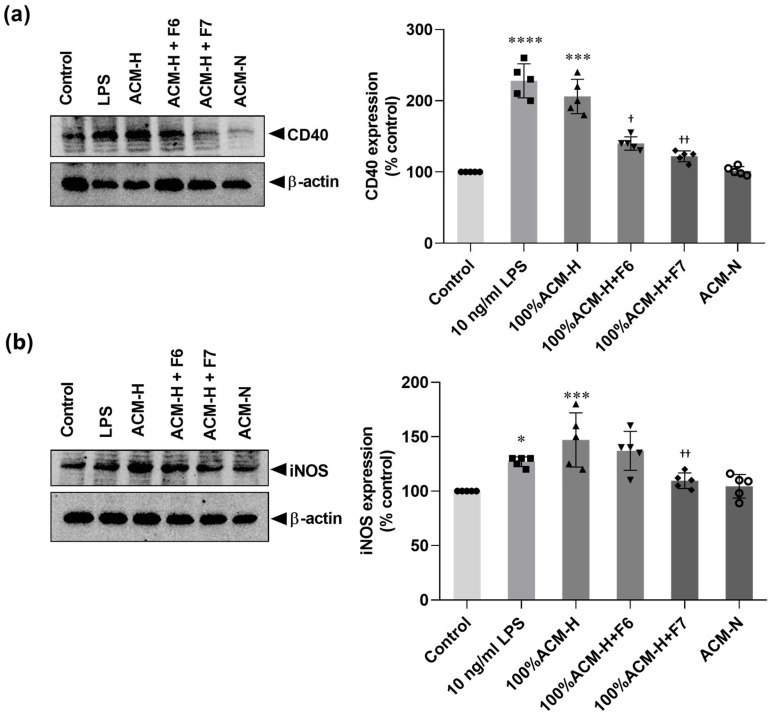
CP fractions suppress ACM-H-induced pro-inflammatory microglial activation. Representative immunoblot images and quantitative analyses of CD40 (**a**) and iNOS (**b**) expression in microglia under control conditions, after stimulation with LPS, ACM-H, ACM-H co-treated with CP seed fractions F6 or F7, and ACM-N. Protein bands were detected by immunoblotting, and expression levels were quantified by densitometric analysis. Data were normalized to β-actin and expressed as percentages of control. Data are presented as mean ± SEM from independent biological experiments (n = 5). * *p* < 0.05, *** *p* < 0.001, **** *p* < 0.0001 vs. control; ^†^ *p* < 0.05, ^††^ *p* < 0.01 vs. 100% ACM-H.

The expression of CD40 and iNOS was evaluated by Western blotting (Figure 6; uncropped blots are shown in Figure S3). LPS stimulation markedly increased CD40 protein expression compared with the control group (Figure 6a). Similarly, exposure to ACM-H significantly upregulated CD40 expression to levels comparable to those induced by LPS. Co-treatment with CP fraction F6 attenuated ACM-H-induced CD40 upregulation, whereas fraction F7 produced a stronger suppressive effect, reducing CD40 expression close to control levels. ACM-N did not alter CD40 expression.

A similar pattern was observed for iNOS expression (Figure 6b). ACM-H significantly increased iNOS levels compared with control microglia, whereas LPS induced a moderate increase. Co-treatment with CP fraction F7 significantly suppressed ACM-H-induced iNOS expression, while fraction F6 produced a partial reduction that did not reach statistical significance. ACM-N treatment maintained iNOS expression at levels comparable to control cells. These results show that CP fractions, particularly F7, attenuate ACM-H-induced CD40 and iNOS expression in microglia.

**Figure 7:**
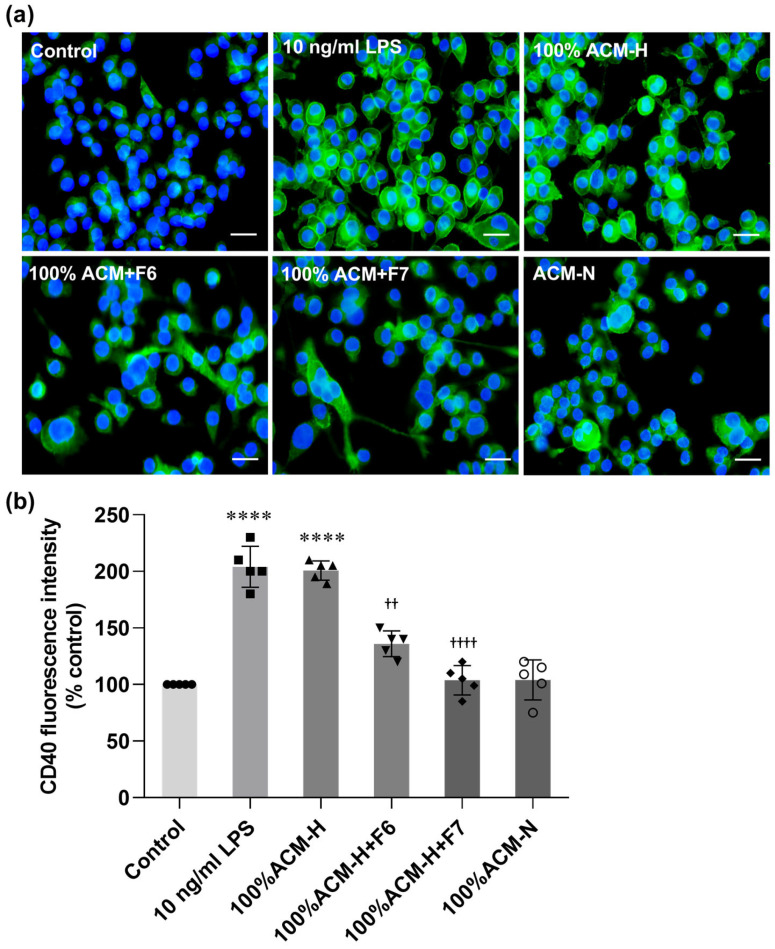
CP fractions suppress ACM-H-induced CD40 upregulation in microglia. Representative immunofluorescence images (**a**) and quantitative analysis (**b**) of CD40 expression in microglia under control conditions, after stimulation with LPS, ACM-H, ACM-H co-treated with CP seed fractions F6 or F7, and ACM-N. CD40 immunoreactivity was detected by immunofluorescence staining, and fluorescence intensity was quantified and normalized to control (% of control). Data are presented as mean ± SEM from independent biological experiments (n = 5). Scale bar = 50 µm. **** *p* < 0.0001 vs. control; ^††^ *p* < 0.01, ^††††^ *p* < 0.0001 vs. 100% ACM-H.

Immunofluorescence analysis confirmed modulation of CD40 expression under different conditions (Figure 7a). Control microglia showed weak CD40 immunoreactivity with a ramified morphology, whereas LPS and ACM-H increased CD40 fluorescence intensity and were associated with enlarged cell bodies and reduced process complexity. Co-treatment with CP fractions attenuated ACM-H-induced CD40 staining, with F7 producing a greater reduction than F6. Microglia treated with ACM-N displayed low CD40 immunoreactivity. Quantitative analysis (Figure 7b) supported these findings, showing increased CD40 levels following LPS and ACM-H treatment and significant reduction after co-treatment with CP fractions. These results show that CP fractions, particularly F7, suppress ACM-H-induced CD40 upregulation in microglia.

### 2.7. CP Fractions Attenuate ACM-H-Induced Pro-Inflammatory Cytokine Release in Microglia

**Figure 8:**
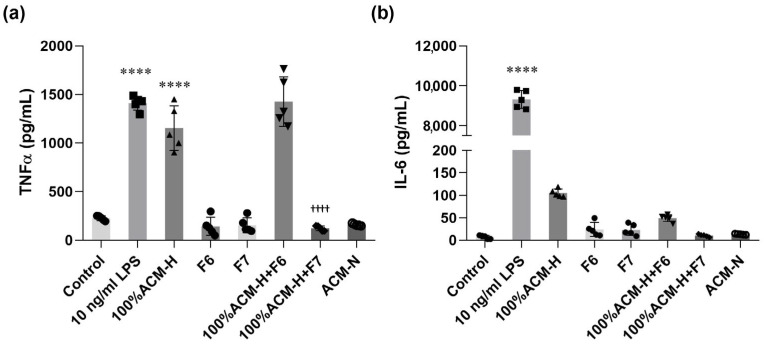
CP fractions suppress ACM-H-induced pro-inflammatory cytokine release from microglia. The concentrations of pro-inflammatory cytokines in culture media were quantified 24 h after treatment using a multiplex bead-based immunoassay. (**a**) TNF-α levels measured under control conditions, following stimulation with LPS (10 ng/mL), ACM-H, treatment with CP seed fractions F6 or F7 alone, ACM-H co-treated with CP fractions F6 or F7, and ACM-N. (**b**) IL-6 levels measured under the same experimental conditions. Data are presented as mean ± SEM from independent biological experiments (n = 5). **** *p* < 0.0001 vs. control; ^††††^ *p* < 0.0001 vs. 100% ACM-H.

Pro-inflammatory cytokines, including tumor necrosis factor-α (TNF-α) and interleukin-6 (IL-6), were quantified in culture media 24 h after treatment using a multiplex bead-based immunoassay (ProcartaPlex™) analyzed on a Luminex™ platform. Under basal conditions, TNF-α levels were low in control cultures (Figure 8a). Stimulation with LPS (10 ng/mL) increased TNF-α release, whereas exposure to ACM-H also significantly elevated TNF-α levels. Co-treatment with CP fraction F7 significantly reduced ACM-H-induced TNF-α production, whereas fraction F6 did not produce a significant effect. Microglia treated with ACM-N maintained TNF-α levels. A similar pattern was observed for IL-6 secretion (Figure 8b). Both LPS and ACM-H increased IL-6 levels compared with control cultures. Co-treatment with CP fractions reduced IL-6 levels relative to ACM-H alone, with F7 showing a greater effect than F6. IL-6 concentrations in the ACM-N group remained low, and treatment with F6 or F7 alone did not significantly affect IL-6 levels.

### 2.8. CP Fractions Restore Microglial Phagocytic Function Following ACM-H-Induced Inflammatory Activation

**Figure 9:**
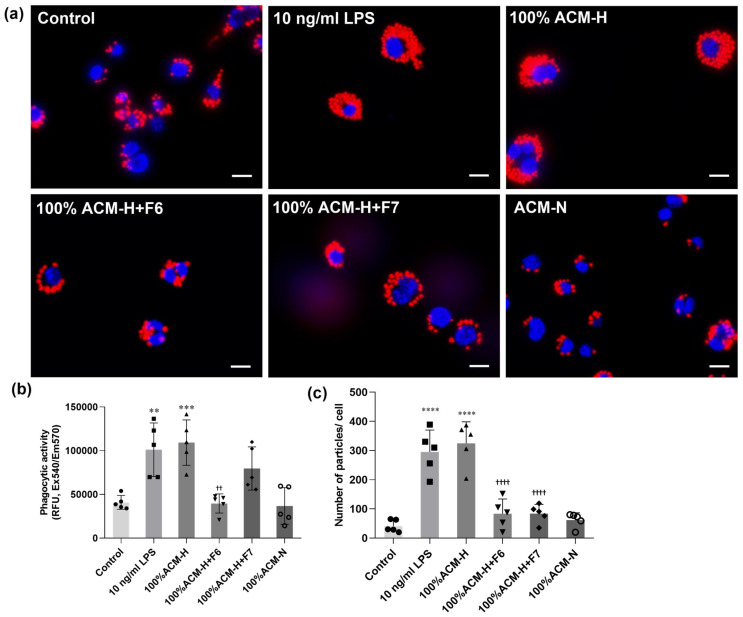
CP fractions attenuate astrocyte-conditioned medium-induced microglial phagocytic activity. (**a**) Representative fluorescence images of BV-2 microglia treated with control medium, lipopolysaccharide (LPS, 10 ng/mL), ACM-H, ACM-H in the presence of CP fractions F6 or F7, and ACM-N. Red fluorescence indicates zymosan-associated signal used to evaluate phagocytic activity. Scale bar = 50 µm. (**b**) Quantification of phagocytic activity measured as fluorescence intensity (RFU; Ex 540/Em 570 nm). (**c**) Quantification of phagocytosis expressed as the number of fluorescent particles per cell. Data are presented as mean ± SEM from independent biological experiments (n = 5). ** *p* < 0.01, *** *p* < 0.001, **** *p* < 0.0001 vs. control; ^††^ *p* < 0.01, ^††††^ *p* < 0.0001 vs. 100% ACM-H.

Microglial phagocytic activity was assessed using a red zymosan-based assay (Figure 9). Cells were maintained under control conditions, stimulated with LPS or ACM-H, and co-treated with CP fractions F6 or F7. ACM-N served as a physiological comparison group. Representative immunofluorescence images (Figure 9a) showed low basal zymosan-associated fluorescence under control conditions and ACM-N exposure. In contrast, LPS and ACM-H stimulation markedly increased intracellular red zymosan signal intensity. Co-treatment with CP fractions F6 or F7 visibly attenuated zymosan-associated fluorescence compared with the ACM-H group. Microglia exposed to ACM-H co-treated with F6 or F7 exhibited reduced red fluorescence intensity and fewer intracellular zymosan aggregates, approaching levels observed under control or ACM-N conditions. Quantitative analysis (Figure 9b,c) confirmed these findings, showing increased zymosan-associated fluorescence signal following LPS and ACM-H treatment and significant reduction after co-treatment with CP fractions.

## 3. Discussion

The present study investigated the effects of high-polarity fractions from CP seeds on neuroinflammatory responses, with a focus on astrocyte–microglia interactions. Using an in vitro sterile inflammation model, ACM-H treatment was found to induce microglial activation, as evidenced by morphological changes, increased expression of CD40 and iNOS, and elevated production of TNF-α and IL-6, along with altered phagocytic activity. These findings are consistent with previous reports showing that stressed astrocytes can modulate microglial activation and neuroinflammatory responses. In the present experimental context, the ACM-H model represents the effects of transient oxidative stress followed by a recovery phase, resulting in a stress-associated astrocyte secretory profile. Treatment with CP seed fractions attenuated several ACM-H-induced responses. Fraction F7 tended to show greater effects on CD40 and iNOS expression and cytokine production, whereas F6 exhibited comparable activity across several assays and appeared to have a more pronounced effect on phagocytic responses. Untargeted metabolomic profiling indicated the presence of diverse alkaloid- and terpenoid-related metabolites in these fractions. While differences in metabolite composition were observed between F6 and F7, the current data do not establish direct causal relationships between specific compounds and the observed biological effects, and further targeted studies will be required to clarify these associations.

Astrocytes play a key role in shaping microglial inflammatory responses through the release of soluble mediators, including cytokines, reactive oxygen species, ATP, and other damage-associated signals [16]. In the present study, ACM-H induced microglial activation, whereas ACM-N did not significantly alter microglial morphology or inflammatory marker expression. The use of H_2_O_2_ to induce astrocyte stress is well-established in experimental models. However, the concentration and exposure duration critically determine the nature and severity of the cellular response. Previous studies have demonstrated that high concentrations of H_2_O_2_ can induce pronounced oxidative injury and inflammatory activation in astrocytes. For example, exposure of C6 astrocytes to 1 mM H_2_O_2_ for 30 min significantly increased oxidative stress, upregulated iNOS, promoted TNF-α release, and disrupted mitochondrial function [17]. In contrast, moderate concentrations of H_2_O_2_ can induce controlled oxidative stress without causing immediate cell death. For example, exposure of rat astrocytes to 100 μM H_2_O_2_ for 10 min has been shown to trigger intracellular signaling responses without inducing apoptosis, enabling investigation of stress-induced astrocytic activation and membrane alterations [18,19]. Oxidative stress has also been reported to disrupt astrocytic metabolic homeostasis and cellular energy balance, including disturbances in energy metabolism and osmotic regulation, leading to prolonged functional alterations even after the oxidative stimulus is removed [20]. Consistent with these observations, astrocytes in the present study were transiently exposed to H_2_O_2_ and subsequently maintained in fresh medium to generate conditioned medium reflecting stress-associated astrocytic responses. H_2_O_2_-induced oxidative stress can elicit dose- and time-dependent astrocytic responses, ranging from cytotoxic effects to controlled activation of intracellular signaling and metabolic changes. These responses may contribute to the release of signaling molecules that influence microglial activity. Accordingly, this approach provides an in vitro system for examining astrocyte-driven inflammatory signaling. Although the H_2_O_2_ concentration used in this study is higher than that reported in previous studies, the exposure was transient and followed by a recovery phase. This design was intended to generate conditioned medium enriched in astrocyte-derived stress signals rather than to induce sustained cytotoxic effects in astrocytes.

Neuroinflammatory responses can be triggered by both pathogen-associated and damage-associated signals. While lipopolysaccharide (LPS) is widely used to induce inflammatory activation through TLR4–NF-κB signaling [21,22], neurodegenerative conditions are more commonly associated with sterile inflammation driven by endogenous signals released from stressed or damaged cells [21]. In this context, astrocytes exposed to oxidative stress can release mediators that influence microglial activation [23]. Accordingly, conditioned medium from stressed astrocytes provides an in vitro approach to model astrocyte-driven inflammatory signaling. However, the molecular composition of ACM-H was not directly characterized in this study, and the specific mediators responsible for microglial activation remain undefined. Therefore, the observed effects should be interpreted within the context of a functional model, and further studies are required to identify the components contributing to these responses.

Microglial activation is associated with increased expression of pro-inflammatory signaling pathways and activation markers such as CD40. CD40 signaling has been linked to the production of inflammatory mediators, including TNF-α, IL-6, and iNOS [24,25]. In the present study, ACM-H increased CD40 expression together with elevated cytokine and iNOS levels, supporting activation of pro-inflammatory microglial responses. These findings are consistent with previous reports of increased CD40 and inflammatory mediator expression in activated microglia [25,26]. LPS and ACM-H represent distinct models of microglial activation. LPS induces responses through PAMP-mediated signaling, whereas ACM-H reflects astrocyte-derived stress signals associated with sterile inflammation. Differences in cytokine magnitude between these models are therefore expected and do not necessarily indicate differences in biological relevance. Morphological assessment based on phase-contrast imaging provides a semiquantitative evaluation and lacks the specificity of fluorescence-based approaches. Accordingly, morphological changes should be interpreted together with additional indicators of microglial activation, including CD40 and iNOS expression, cytokine production, and phagocytic activity. Modulation of CD40-associated signaling may contribute to the regulation of microglial activation under inflammatory conditions. In addition to cytokine production, microglia exhibit functional responses such as phagocytosis. However, the zymosan-based assay used in this study provides a semiquantitative measure and does not distinguish between particle adhesion and internalization. Therefore, changes in fluorescence signal should be interpreted with caution, and further studies using more specific approaches are required.

Phytochemical investigations of CP have revealed a diverse array of secondary metabolites associated with a broad range of reported pharmacological activities [11,12]. Previous studies have described the presence of multiple classes of constituents, including monoterpenes, sesquiterpenes, diterpenoids, triterpenoids, alkaloids, flavonoids, fatty acids, and steroids [27], which have been reported to exhibit antioxidant [28], anti-inflammatory [29], neuroprotective [13], and cognition-related effects [30]. Consistent with these reports, untargeted metabolite profiling in the present study indicated that the high-polar fractions contain structurally diverse metabolites, including alkaloid- and terpenoid-related features. These observations provide a compositional context for interpreting the biological responses observed in the astrocyte–microglia model. While variation in metabolite composition was observed between F6 and F7, the current data do not establish direct causal relationships between specific compounds and the observed attenuation of microglial inflammatory responses. Rather, the findings suggest that the overall metabolite composition of the fractions may be associated with their observed biological effects. Previous studies have reported that various alkaloid and terpenoid derivatives can modulate inflammatory signaling pathways and oxidative stress responses in neural cells. However, further targeted studies will be required to identify the specific constituents responsible for the effects observed in the present study. Therefore, the metabolomic characteristics of these fractions provide a compositional context for interpreting their observed effects on astrocyte–microglia interactions. Collectively, these findings suggest that CP-derived metabolites modulate neuroinflammatory responses in the present experimental model; however, these observations are limited to an in vitro system and require further validation in more complex models.

## 4. Materials and Methods

### 4.1. Preparation of CP Seed Fractions

CP seeds were kindly provided by the Queen Sirikit Botanic Garden, Botanical Garden Organization, Ministry of Natural Resources and Environment, Mae Rim, Chiang Mai, Thailand. Dried seeds were powdered and extracted with 95% ethanol for 72 h at 25 °C. The crude extract was concentrated under reduced pressure and subjected to silica gel column chromatography. Fractionation was performed using a stepwise gradient elution system of increasing polarity, consisting of hexane–ethyl acetate mixtures (100:0 to 0:100, *v*/*v*), followed by methanol (100%). Eluted subfractions were collected sequentially and combined based on thin-layer chromatography (TLC) profiles and retention factor (Rf) similarity, yielding seven major fractions (F1–F7) arranged according to increasing polarity. Fraction F6 was obtained from intermediate-to-high polarity eluents, including hexane–ethyl acetate mixtures ranging from 50:50 to 10:90 (*v*/*v*), as well as 100% ethyl acetate. In contrast, fraction F7 was collected as the most polar fraction using 100% methanol. Although both F6 and F7 are categorized as high-polar fractions, they were obtained from distinct solvent systems and exhibited different chromatographic characteristics based on TLC analysis. F6 and F7 exhibited distinct TLC profiles with different Rf distributions, supporting their separation into chemically distinct fractions. In the present study, these two fractions were selected for subsequent chemical characterization and biological evaluation.

### 4.2. LC–MS/MS-Based Untargeted Metabolite Profiling

Untargeted metabolite profiling of CP seed fractions was conducted by U2Bio Thailand using liquid chromatography–tandem mass spectrometry (LC–MS/MS). Approximately 10 mg of each sample (F6 and F7 fractions) was extracted with 1000 µL of 70% methanol containing sulfadimethoxine (50 ng/mL) as an internal standard. Samples were vortexed and centrifuged at 14,000 rpm for 10 min, and the resulting supernatants were collected and transferred to LC vials for analysis.

#### 4.2.1. Liquid Chromatography Conditions

Chromatographic separation was performed on a Poroshell 120 EC-C18 column (2.1 × 100 mm, 2.7 µm; Agilent Technologies) maintained at 50 °C. The injection volume was 10 µL, and the flow rate was set to 0.4 mL/min. The mobile phases consisted of (A) 0.1% formic acid in water and (B) 0.1% formic acid in acetonitrile, optimized for electrospray ionization (ESI). The LC gradient program was as follows: 0–0.5 min, 100% A; 0.5–10.5 min, linear gradient to 55% B; 10.5–12.5 min, 75% B; 12.5–14.0 min, 100% B; 14.0–17.0 min, held at 100% B; 17.0–17.5 min, returned to initial conditions; and equilibrated at 100% A until 20.0 min prior to the next injection.

#### 4.2.2. Mass Spectrometry Conditions and Quality Control

Mass spectrometric analysis was performed using an Agilent 6545XT Q-TOF mass spectrometer (Agilent Technologies, Santa Clara, CA, USA) equipped with an electrospray ionization (ESI) source operated in both positive and negative ion modes. Source parameters were set as follows: drying gas temperature, 325 °C; drying gas flow, 13 L/min; sheath gas temperature, 275 °C; sheath gas flow, 12 L/min; nebulizer pressure, 45 psi; and capillary voltage, 4000 V (positive mode) and 3000 V (negative mode).

MS1 spectra were acquired over an *m*/*z* range of 40–1700, and MS/MS spectra were acquired over an *m*/*z* range of 25–1000 using data-dependent acquisition. Collision energy was set at 20 eV (positive mode) and 10 eV (negative mode). The acquisition rate was 3.35 spectra/s, with a maximum of 10 precursor ions selected per cycle and a precursor intensity threshold of 5000 counts. Continuous mass calibration was performed using reference ions at *m*/*z* 121.0509 and 922.0098 (positive mode), and *m*/*z* 112.9856 and 1033.9881 (negative mode).

Given that only two samples were analyzed, each as a single injection in a continuous sequence, pooled QC samples were not included. Instrument performance and data quality were instead monitored using internal standard responses, blank runs to assess background signals, and mass calibration to ensure mass accuracy and system stability throughout the analysis. Mass calibration reports demonstrating instrument performance are provided in the Supplementary Information.

#### 4.2.3. Data Processing and Metabolite Annotation

Raw LC–MS/MS data were processed using U2Bio’s standard untargeted metabolomics workflow, including peak detection (feature extraction), retention time alignment across samples, and feature filtering. Background signals were evaluated using blank samples, and features with high blank intensity were excluded to minimize non-specific signals. Signal intensities were normalized to the internal standard and total ion signal to reduce technical variation across samples.

Putative metabolite identification was performed based on accurate mass measurement, chromatographic behavior, and MS/MS spectral matching against public spectral databases. Metabolite annotation confidence corresponded to level 2–3 identification according to commonly accepted metabolomics reporting standards. No metabolite identification was confirmed using authentic reference standards.

A predefined quality-filtering pipeline was applied to the positive ion dataset to enhance analytical robustness. Metabolite features were retained only when all of the following criteria were satisfied: (1) total identification score ≥ 1.3, (2) average signal-to-noise ratio (S/N) ≥ 12, (3) blank intensity ≤ 10,000, and (4) normalized intensity ≥ 1.4 × 10^6^ in either the F6 or F7 fraction. These thresholds were selected to minimize false-positive annotations and ensure reliable structural assignment.

### 4.3. Cell Culture

#### 4.3.1. Astrocyte Culture and Induction of Stress-Conditioned Medium

CTX TNA2 astrocytes (rat cortical astrocyte cell line) were maintained in Dulbecco’s Modified Eagle Medium (DMEM, high glucose) supplemented with 10% fetal bovine serum (FBS) and 1% penicillin–streptomycin under standard culture conditions (37 °C, 5% CO_2_). To induce oxidative stress, astrocytes were exposed to hydrogen peroxide (H_2_O_2_; 200 µM, approximately 5.9 mM) for 1 h. Following exposure, the H_2_O_2_-containing medium was removed, and cells were washed and replaced with fresh complete DMEM. Astrocytes were then maintained for an additional 72 h to allow recovery and the accumulation of stress-induced secreted factors. The resulting conditioned medium was collected and referred to as ACM-H.

ACM-H was collected and centrifuged to remove cellular debris. The supernatant was aliquoted and stored at −80 °C. For concentration, frozen samples were positioned at a 45° angle for 24 h prior to lyophilization and subsequently subjected to freeze-drying using an Alpha 2–4 LSCplus freeze dryer (Martin Christ, Osterode am Harz, Germany) for 2–3 days until complete dryness was achieved. Lyophilized samples were stored at −20 °C and reconstituted in appropriate culture medium to obtain final concentrations corresponding to 50% or 100% ACM-H for subsequent experiments.

#### 4.3.2. Microglial Culture and Treatment

BV-2 microglial cells were maintained in RPMI-1640 medium supplemented with 10% fetal bovine serum (FBS) and 1% penicillin–streptomycin under standard culture conditions (37 °C, 5% CO_2_). For experimental stimulation, BV-2 cells were first exposed to 50% or 100% ACM-H for 24 h. In parallel, microglia treated with ACM-N served as a non-inflammatory control. After the initial 24 h stimulation period, the medium was replaced, and cells were subsequently treated with CP seed fractions F6 or F7 for an additional 24 h. Control groups received corresponding complete RPMI-1640 medium without conditioned medium or CP fractions.

### 4.4. Phase-Contrast Microscopy and Morphological Assessment

BV-2 microglial cells were seeded in 24-well plates (5 × 10^4^ cells/well) and allowed to adhere overnight. Cells were treated with lipopolysaccharide (LPS, 10 ng/mL), ACM-H (50% or 100%), ACM-H combined with CP seed fractions F6 or F7, ACM-N, or culture medium alone (control) for 24 h. Cellular morphology was assessed by phase-contrast microscopy (model, manufacturer) at 20× magnification. For each condition, images were captured from at least five randomly selected fields per well in three independent experiments. Morphometric analysis (cell body area, total process length, and ramification index) was performed using ImageJ (version 1.53, NIH, Bethesda, MD, USA), with at least 30 cells analyzed per condition in a blinded manner.

### 4.5. Cytokine Quantification by ProcartaPlex Multiplex Immunoassay

The concentrations of TNF-α and IL-6 in culture supernatants were quantified using the Mouse ProcartaPlex™ Mix&Match 2-plex kit (Thermo Fisher Scientific, Waltham, MA, USA) according to the manufacturer’s protocol. After 24 h of treatment, culture media were collected and centrifuged to remove cellular debris. Samples were incubated with magnetic capture beads specific for TNF-α and IL-6, followed by incubation with biotinylated detection antibodies and streptavidin–phycoerythrin (PE). Data acquisition was performed using a Luminex™ xMAP^®^ platform, and cytokine concentrations were calculated from standard curves generated with recombinant standards. Results were expressed as pg/mL.

### 4.6. Western Blot Analysis

Total cellular protein was extracted from BV-2 microglial cells using RIPA lysis buffer supplemented with protease inhibitors. Equal amounts of protein were resolved by SDS–polyacrylamide gel electrophoresis (SDS–PAGE) and transferred onto polyvinylidene fluoride (PVDF) membranes. Membranes were blocked with One-Step Western Blocking Buffer (Energesis Biochemical, Taipei, Taiwan) for 1 h at 25 °C and subsequently incubated overnight at 4 °C with the following primary antibodies: rabbit anti-CD40 (EPR18005-35, Abcam, Cambridge, UK), rabbit anti-iNOS (ab3523, Abcam), and mouse anti-β-actin (ab6276, Abcam) as a loading control.

After washing with TBST, membranes were incubated for 1 h at 25 °C with horseradish peroxidase (HRP)-conjugated goat anti-rabbit IgG or goat anti-mouse IgG secondary antibodies, as appropriate. Protein bands were detected using enhanced chemiluminescence (ECL) reagents and visualized with a Gel Doc imaging system (Bio-Rad Laboratories, Hercules, CA, USA). Densitometric analysis was performed using ImageJ software (version 1.53, NIH, Bethesda, MD, USA), and target protein expression levels were normalized to β-actin. A prestained protein ladder was used as a molecular weight marker and imaged on the membrane after transfer.

### 4.7. Immunofluorescence Staining

BV-2 microglial cells were seeded onto sterile coverslips and treated under the indicated experimental conditions. Following treatment, cells were fixed with 4% paraformaldehyde for 15 min at room temperature and washed with phosphate-buffered saline (PBS). Cells were permeabilized with 0.1% Triton X-100 in PBS for 10 min and blocked with blocking buffer for 1 h at room temperature to minimize nonspecific binding. Cells were incubated overnight at 4 °C with rabbit anti-CD40 primary antibody (EPR18005-35, Abcam, Cambridge, UK) diluted 1:500 in blocking buffer. After washing with PBS, cells were incubated for 1 h at room temperature in the dark with FITC-conjugated secondary antibody (Abcam, Cat. No. ab6717). Nuclei were counterstained with Hoechst 33342 for visualization. Coverslips were mounted using antifade mounting medium, and fluorescence images were captured using an Olympus BX53 fluorescence microscope (Olympus Corporation, Tokyo, Japan) under identical exposure settings across all experimental groups. Quantitative analysis of CD40 fluorescence intensity was performed using ImageJ software (version 1.53, NIH, Bethesda, MD, USA).

### 4.8. Microglial Phagocytic Activity

Microglial phagocytic activity was assessed using a Red Zymosan Phagocytosis Assay Kit (ab234054; Abcam, Cambridge, UK) according to the manufacturer’s instructions with minor modifications. BV-2 microglial cells were seeded in 24-well plates containing glass coverslips (for immunofluorescence imaging) at a density of 1 × 10^5^ cells/well and allowed to adhere overnight. Cells were then assigned to the following experimental groups: control, LPS-stimulated, ACM-H (astrocyte-conditioned medium from H_2_O_2_-treated astrocytes), ACM-N, and ACM-H co-treated with CP seed fractions F6 or F7. After treatment, cells were incubated with Red Zymosan Bioparticles™ at the recommended concentration (as provided by the kit) for 1–2 h at 37 °C in a humidified 5% CO_2_ incubator to allow phagocytosis. Following incubation, cells were washed thoroughly with cold PBS to remove non-associated particles. To quench extracellular fluorescence, a quenching solution provided in the kit was applied according to the manufacturer’s protocol. For fluorescence imaging, cells on coverslips were fixed with 4% paraformaldehyde for 15 min at room temperature, counterstained with Hoechst 33342 for nuclear visualization, and mounted using antifade mounting medium. Images were captured using a fluorescence microscope under identical exposure settings for all groups. For quantitative analysis, fluorescence intensity was measured using a microplate reader at Ex/Em = 540/570 nm. Relative phagocytic activity was calculated after background subtraction and expressed relative to the control group. Phagocytic activity was further quantified as the number of fluorescent particles per cell based on image analysis to account for differences in cell number. Image analysis was performed using consistent thresholding criteria, and all analyses were conducted in a blinded manner. The zymosan-based assay provides a semiquantitative assessment and does not distinguish between particle internalization and surface adhesion.

### 4.9. Statistical Analysis

All data are expressed as the mean ± standard error of the mean (SEM) from at least three independent biological experiments. Statistical comparisons among multiple groups were performed using one-way analysis of variance (ANOVA) followed by Tukey’s post hoc test for multiple comparisons to control for type I error. No pairwise t-tests were used in this study. Statistical analyses were conducted using GraphPad Prism software (version 8.0; GraphPad Software, San Diego, CA, USA). A *p* value < 0.05 was considered statistically significant.

## 5. Conclusions

In summary, LC–MS/MS profiling showed that the F6 and F7 fractions of *Celastrus paniculatus* (CP) seeds contain diverse secondary metabolites, including alkaloid- and terpenoid-related compounds. Functionally, these fractions modulated microglial responses under ACM-H-induced inflammatory conditions. CP fractions attenuated morphological changes, reduced CD40 and iNOS expression, and decreased the release of pro-inflammatory cytokines. In addition, CP fractions reduced phagocytic activity following inflammatory stimulation. Collectively, these findings suggest that CP seed fractions can modulate microglial activation and inflammatory signaling in this in vitro model. Further studies are required to identify the active constituents and to determine their relevance in more complex experimental systems.

## Figures and Tables

**Table 1:** Identified metabolites in the bioactive fractions (F6 and F7) of CP seeds classified by chemical class.

No.	Putatively Annotated Metabolite	Chemical Class	Major Chemical Group
1.	Reserpic acid *	Corynanthean-type alkaloids	Alkaloids
2.	Gelsemine *	Gelsemium alkaloids	
3.	Corynoxeine	Indolizidines	
4.	Dehydrocorydalin	Protoberberine alkaloids	
5.	Catharanthine	Ibogan-type alkaloids	
6.	Magnoflorine	Aporphines	
7.	Belladonnine	Tropane-related alkaloids	
8.	Columbamine	Protoberberine alkaloids	
9.	Vincarine	Corynanthean-type alkaloids	
10.	Cinchonine	Cinchona alkaloids	
11.	N-(3,4-dimethoxyphenethyl)-1H-indole-3-carboxamide	Indole alkaloids	
12.	Putative indole alkaloid derivative	Indole alkaloids	
13.	Putative terpene lactone * (NCGC00384888-01)	Terpene lactones	Terpenoids
14.	1,6-O,O-diacetylbritannilactone	Terpene lactones	
15.	Ophiopogonoside A	Terpene glycosides	
16.	Putative triterpenoid * (NCGC00384869-01)	Triterpenoids	
17.	Putative terpene glycoside (NCGC00169456-02)	Terpene glycosides	
18.	Putative terpene glycoside (NCGC00384740-01)	Terpene glycosides	
19.	(+)-Costunolide	Sesquiterpene lactones	
20.	Putative diterpenoid (NCGC00169113-03)	Diterpenoids	
21.	Putative acylated terpenoid (NCGC00381245-01)	Terpenoid derivatives	
22.	Phorbol	Diterpenoids	
23.	Ganolactone B	Triterpenoids	
24.	Putative steroid-like triterpenoid (potassium carboxylate)	Triterpenoids	
25.	Precyasterone	Ecdysteroids	
26.	14,15beta-Dihydroxyklaineanone	Diterpenoids	
27.	Putative sesquiterpene glycoside (NCGC00385391-01)	Sesquiterpene glycosides	
28.	Putative geranyl diglycoside	Monoterpene glycosides	
29.	Putative steroidal lactone (NCGC ID)	Steroidal triterpenoids	
30.	Abietin	Abietane-type diterpenoids	
31.	Salidroside	Phenylpropanoid glycosides	Phenolic compounds
32.	Eleutheroside B	Phenylpropanoid glycosides	
33.	Putative chromene (coumarin-like) derivative	Coumarins/Chromene derivatives	
34.	Citrusin (putative; isomer not resolved)	Flavonoid glycosides	Flavonoids
35.	Glabrol	Prenylated flavonoids	

1. Metabolite identification was based on LC–MS/MS spectral matching and database annotation. 2. Compounds labeled as “putative” were assigned based on spectral similarity and literature comparison and were not confirmed with authentic standards. 3. All metabolites were putatively annotated based on accurate mass and MS/MS spectral matching and correspond to MSI level 2. * Selected compounds were used for representative extracted ion chromatogram (XIC) analysis (Figure 2).

## Data Availability

The data could be downloaded from the public databases, and no additional data are available. Further inquiries can be directed to the corresponding author.

## Supplementary Materials

The following supporting information can be downloaded at: https://www.mdpi.com/article/10.3390/ijms27083551/s1.

## Abbreviations

The following abbreviations are used in this manuscript:

ACM	Astrocyte-conditioned medium
ACM-H	Astrocyte-conditioned medium derived from H_2_O_2_-treated astrocytes
ACM-N	Astrocyte-conditioned medium derived from normal astrocytes
CP	*Celastrus paniculatus*
DAMPs	Damage-associated molecular patterns
IL-6	Interleukin-6
iNOS	Inducible nitric oxide synthase
LPS	Lipopolysaccharide
LC–MS/MS	Liquid chromatography–tandem mass spectrometry
TIC	Total ion chromatogram
TNF-α	Tumor necrosis factor alpha
