# Supplementary material for: TLC-Derived High-Polar Fractions of Celastrus paniculatus Seeds Attenuate Astrocyte-Driven Microglial Activation Through Suppression of CD40/iNOS Signaling and Pro-Inflammatory Cytokines
Source: Int J Mol Sci. 2026 Apr 16;27(8):3551. doi: 10.3390/ijms27083551 (PMC13116680; doi:10.3390/ijms27083551)
Supplement: Supplementary file 1 [file ijms-27-03551-s001.zip › ijms-4227845-supplementary Figures.pdf]

Chromatogram (Positive mode)

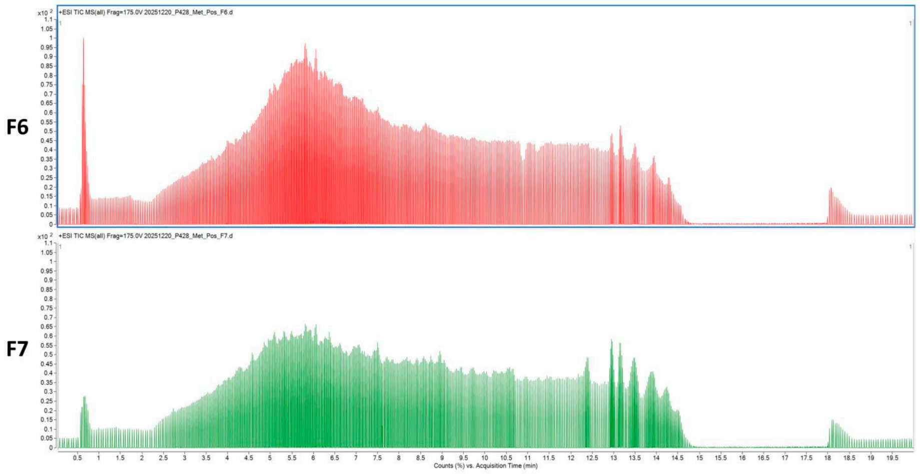

Chromatogram (Negative mode)

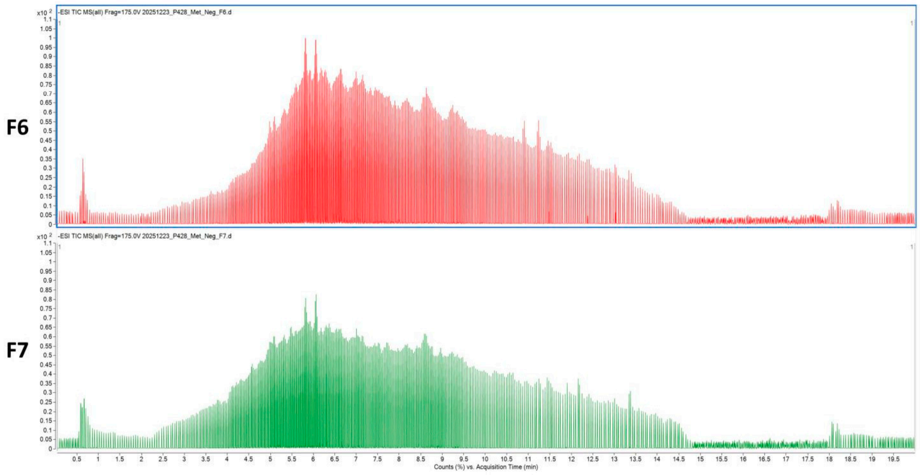

Supplementary Figure S1. Total ion chromatograms (TICs) of *Celastrus paniculatus* seed fractions F6 and F7 obtained by LC–MS/MS analysis.

Chromatographic profiles of the TLC-derived fractions F6 and F7 are shown in both positive ion mode (upper panel) and negative ion mode (lower panel). The chromatograms illustrate the overall ion abundance patterns and retention time distribution of detected metabolites within each fraction. Similar chromatographic patterns were observed between F6 and F7, indicating broadly overlapping metabolite compositions, although differences in peak intensity suggest fraction-dependent variation in relative metabolite abundance.

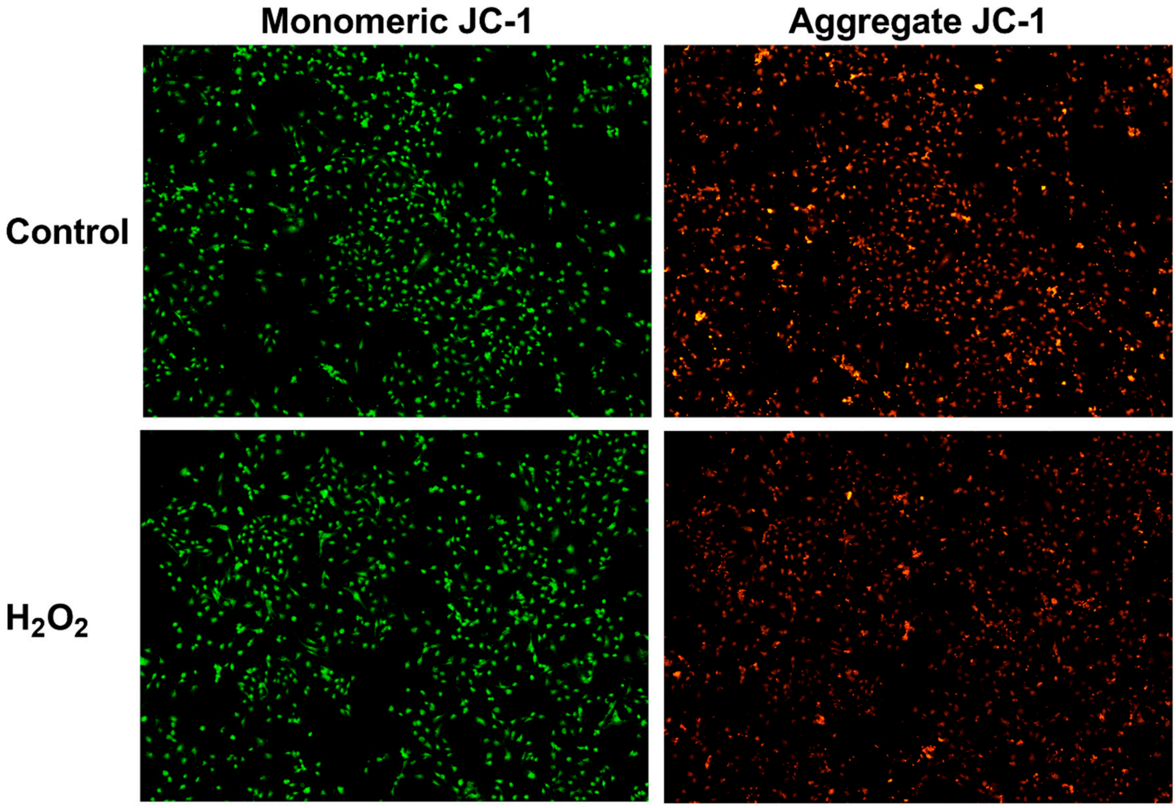

Supplementary Figure S2. Validation of oxidative stress–induced astrocyte activation using JC-1 staining.

Representative JC-1 fluorescence images of CTX TNA2 astrocytes under control conditions and following exposure to H_2O_2 (200 μg/mL) for 1 h. JC-1 staining was used to assess mitochondrial membrane potential (ΔΨm), where red fluorescence indicates JC-1 aggregates in polarized mitochondria, and green fluorescence indicates JC-1 monomers in depolarized mitochondria.

H_2O_2-treated astrocytes exhibited a marked reduction in red fluorescence and an increase in green fluorescence compared with control cells, indicating mitochondrial depolarization and oxidative stress. These results support the induction of a stress-associated astrocyte phenotype used for the generation of conditioned medium (ACM-H).

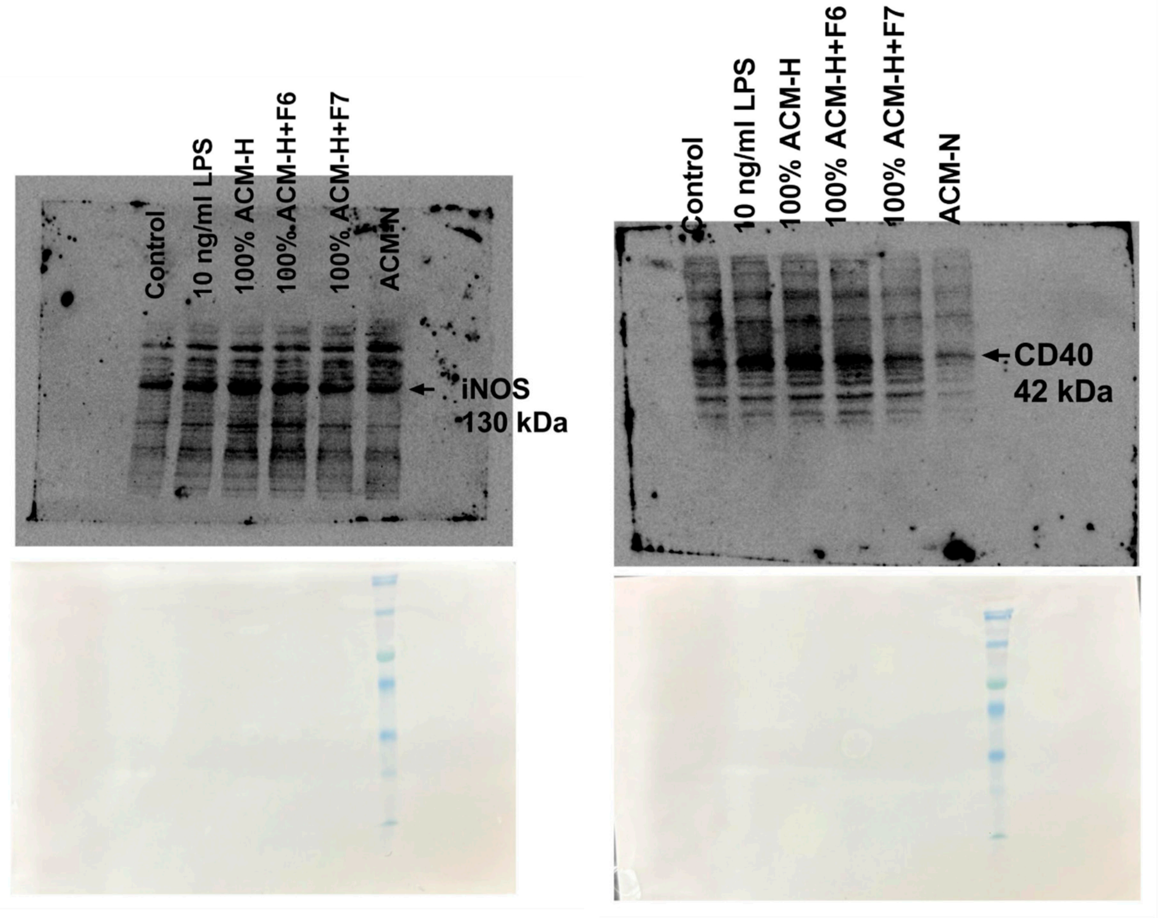

Supplementary Figure S3. Full-length uncropped Western blot images for CD40 and iNOS expression in BV-2 microglia.

(a) Full-length immunoblot of iNOS (~130 kDa) and (b) CD40 (~42 kDa) under the indicated experimental conditions: Control, LPS (10 ng/mL), 100% ACM-H, 100% ACM-H co-treated with F6 or F7, and ACM-N.

Uncropped membranes are shown to demonstrate the specificity of the detected bands and overall protein distribution. Molecular weight markers were obtained from a prestained protein ladder visualized on the same membrane after transfer and are shown below each corresponding blot. Band positions corresponding to target proteins are indicated.

These images correspond to the cropped blots presented in the main figures.
