# Supplementary material for: TLC-Derived High-Polar Fractions of Celastrus paniculatus Seeds Attenuate Astrocyte-Driven Microglial Activation Through Suppression of CD40/iNOS Signaling and Pro-Inflammatory Cytokines
Source: Int J Mol Sci. 2026 Apr 16;27(8):3551. doi: 10.3390/ijms27083551 (PMC13116680; doi:10.3390/ijms27083551)
Supplement: Supplementary file 1 [file ijms-27-03551-s001.zip › table S1.pdf]

Table S1. Annotated metabolites detected in the bioactive fractions (F6 and F7) of *Celastrus paniculatus* seeds by LC–MS/MS analysis.

										normalized intensity	
Alignment ID	verage RT (min	Average m/z	ss error (pp	Metabolite name	Adduct type	Ontology	Total score	S/N average	70%MeOH blank	F6	F7
1619	5.436	285.13336	-11.78	low score: 3-Hexen-1-ol O-b-D-glucopyranoside	[M+Na]+	Fatty acyl glycosides of mono- and disaccharides	1.595	89.11	338.12	38,818,345.24	17,859,955.06
2359	5.176	359.16946	1.53	catharanthine	[M+Na]+	Ibogan-type alkaloids	1.695	113.46	0.00	35,829,506.14	4,912,547.92
2594	6.509	383.20612	-3.68	low score: Histamine-trifluoromethyltoluide	[M+H]+	Trifluoromethylbenzenes	1.408	98.43	0.00	29,781,528.13	24,271,156.50
1850	6.444	309.20905	-9.80	low score: albocycline	[M+H]+	Macrolides and analogues	1.315	98.42	1,355.06	27,870,609.03	17,177,815.14
2820	6.343	405.24814	4.59	low score: (E)-10,10-dimethyl-10a-(3-(pentyloxy)styry	[M+H]+	Indoles and derivatives	1.543	162.01	479.32	22,324,207.51	17,056,984.52
3463	5.518	473.23593	0.13	low score: NCGC00169456-02_C21H38O10_(1R,2R,4a	[M+Na]+	Terpene glycosides	1.481	57.01	447.31	21,971,271.71	4,243,417.28
2190	5.856	343.17322	-9.35	low score: Magnoflorine	[M+H]+	Aporphines	1.549	46.29	110.63	21,455,383.09	2,920,663.36
1620	5.603	285.13382	-13.40	low score: 9-(2,3-dihydroxypropoxy)-9-oxononanoic a	[M+Na]+	Medium-chain fatty acids	1.584	61.81	1,131.15	21,408,627.59	8,799,959.42
3464	5.947	473.23599	0.00	low score: NCGC00169456-02_C21H38O10_(1R,2R,4a	[M+Na]+	Terpene glycosides	1.437	23.02	1,012.88	18,393,222.32	6,759,166.40
3013	7.758	425.25275	-4.09	low score: Caperatic acid	[M+Na]+	Tricarboxylic acids and derivatives	1.304	37.01	527.35	18,158,233.11	8,650,235.29
3542	8.316	481.24097	18.76	low score: Ganolactone B	[M+Na]+	Triterpenoids	1.379	45.47	147.29	17,550,344.47	3,894,592.56
3484	5.133	475.25281	-0.25	low score: (E)-5-hydroxy-N-[3-[5-[3-[[(E)-5-hydroxy-3	[M+Na]+	Alpha amino acids and derivatives	1.373	78.11	282.30	16,117,072.70	12,223,949.81
2005	6.509	325.20108	-3.29	low score: N-((octahydro-1H-quinolizin-1-yl)methyl)-2	[M+Na]+	Quinolizines	1.309	20.78	823.51	16,079,474.24	4,033,204.38
2592	6.091	383.20596	-15.53	low score: Corynoxeine	[M+H]+	Indolizidines	1.365	56.57	566.69	15,483,282.97	10,264,972.10
2590	5.557	383.20395	1.98	low score: Histamine-trifluoromethyltoluide	[M+H]+	Trifluoromethylbenzenes	1.417	75.60	9.38	15,367,797.91	2,061,298.58
1618	3.464	285.13193	-6.77	low score: 9-(2,3-dihydroxypropoxy)-9-oxononanoic a	[M+Na]+	Medium-chain fatty acids	1.586	57.42	7.48	14,748,010.94	3,677,039.18
1593	3.988	283.11551	-1.84	low score: MEVALOLACTONE	[2M+Na]+	Delta valerolactones	1.473	19.37	337.57	14,354,726.75	4,739,670.21
2729	5.060	397.18329	-8.31	low score: 14,15beta-Dihydroxyklaineanone	[M+H]+	Quassinoids	1.493	105.38	0.00	13,101,033.79	1,218,190.77
1762	3.401	301.12546	-18.13	low score: Salidroside	[M+H]+	O-glycosyl compounds	1.410	17.42	50.91	12,438,590.03	3,392,458.89
2362	5.612	359.17477	-13.25	catharanthine	[M+Na]+	Ibogan-type alkaloids	1.710	88.76	214.35	12,366,964.41	1,879,676.33
2208	4.478	345.15286	-8.32	gelsemine	[M+Na]+	Gelsemium alkaloids	1.553	55.89	124.92	12,198,419.73	1,087,942.11
2409	7.485	365.19522	-3.34	NCGC00169827-03!(2E,4E)-12-hydroxy-13-(hydroxym	[M+Na]+	Long-chain fatty acids	1.575	68.57	829.16	12,100,366.29	1,828,939.80
2003	4.451	325.16248	-7.63	low score: N-(3,4-dimethoxyphenethyl)-1H-indole-3-c	[M+H]+	Dimethoxybenzenes	1.487	27.09	214.04	11,511,898.09	400,592.00
4078	13.524	547.38574	-1.33	low score: Arginine conjugated cholic acid	[M-H2O+H]+	Glycinated bile acids and derivatives	1.508	275.09	3,962.90	11,106,268.79	9,179,597.15
2051	5.102	329.15750	1.85	low score: Propentofylline	[M+Na]+	Xanthines	1.429	55.05	0.00	10,821,779.26	3,034,618.97
3704	6.465	501.26932	1.34	low score: Moroidin-[QLLVWRAH]	[M+2H]+	Oligopeptides	1.334	66.06	764.84	10,772,307.34	10,437,184.55
1596	4.649	283.11777	-9.82	low score: MEVALOLACTONE	[2M+Na]+	Delta valerolactones	1.323	65.68	35.99	10,671,793.44	3,227,899.66
3163	7.168	441.24814	-18.47	low score: Ophiopogonoside A	[M+Na]+	Terpene glycosides	1.502	55.73	832.27	9,874,360.76	9,142,057.92
2946	5.189	417.21106	-17.47	6-[[5-(hydroxymethyl)-2,5,8a-trimethyl-1,4,4a,6,7,8-h	[M+K]+	Oxepanes	1.442	46.37	1,130.35	9,789,797.57	10,025,992.38
2191	5.314	343.17352	-10.23	low score: Magnoflorine	[M+H]+	Aporphines	1.501	71.28	0.00	9,502,828.02	3,573,088.84
3466	5.737	473.23718	-2.51	NCGC00385391-01_C21H38O10_2-[2,3,8-Trihydroxy-8	[M+Na]+	Terpene glycosides	1.542	12.74	40.98	9,327,024.78	4,564,115.92
2360	5.397	359.17004	-0.08	catharanthine	[M+Na]+	Ibogan-type alkaloids	1.692	78.21	7.21	8,501,457.61	1,814,448.25
2210	4.880	345.15363	18.46	gelsemine	[M+Na]+	Gelsemium alkaloids	1.554	80.25	67.78	8,410,116.29	4,214,152.31
2193	6.077	343.17508	-14.77	low score: Magnoflorine	[M+H]+	Aporphines	1.621	41.72	1,409.89	8,256,839.83	1,071,446.05
2192	6.300	343.17398	-11.57	low score: Magnoflorine	[M+H]+	Aporphines	1.492	106.95	273.22	8,178,680.97	1,577,527.44
2947	5.655	417.21115	-17.69	6-[[5-(hydroxymethyl)-2,5,8a-trimethyl-1,4,4a,6,7,8-h	[M+K]+	Oxepanes	1.426	48.07	111.84	7,786,720.35	3,269,929.40
2429	4.857	367.17371	-10.08	low score: Dehydrocorydalin	[M+H]+	Protoberberine alkaloids and derivatives	1.611	57.89	0.00	7,065,901.40	5,640,150.55
2270	7.477	351.18140	-4.02	low score: 1,6-O,O-diacetylbritannilactone	[M+H]+	Terpene lactones	1.419	372.37	164.34	6,841,668.64	21,429,528.18
1616	3.692	285.13129	-4.52	low score: 3-Hexen-1-ol O-b-D-glucopyranoside	[M+Na]+	Fatty acyl glycosides of mono- and disaccharides	1.471	32.93	495.15	6,740,386.12	3,099,851.40
1436	5.793	267.15659	7.19	low score: Sambucinol	[M+H]+	Oxepanes	1.627	133.00	3,239.06	6,573,423.35	4,734,101.68
3279	7.066	453.20984	-3.38	low score: Fluocinolone acetonide	[M+H]+	21-hydroxysteroids	1.405	39.30	20.43	6,481,124.23	2,814,378.52
2358	4.164	359.16837	4.57	catharanthine	[M+Na]+	Ibogan-type alkaloids	1.763	25.75	309.09	6,320,959.04	5,291,192.66
1805	3.525	305.15811	-3.31	low score: Hexaethylene glycol	[M+Na]+	Polyethylene glycols	1.714	79.57	8,297.47	6,268,271.51	26,999,610.89
1872	5.060	311.14682	-2.64	low score: HISTIDINE	[2M+H]+	Histidine and derivatives	1.362	25.90	100.82	6,258,902.38	1,804,415.28
3861	8.978	521.30872	2.46	low score: Precyasterone	[M+H]+	Withanolides and derivatives	1.429	26.72	98.11	6,241,635.55	931,148.19
3543	7.913	481.24127	18.14	low score: Ganolactone B	[M+Na]+	Triterpenoids	1.425	20.03	0.00	6,215,635.64	506,901.55
2030	5.145	327.14334	-10.18	low score: Citrusin	[M+H]+	Phenolic glycosides	1.442	15.56	7.51	6,070,290.93	4,111,072.84
2818	8.267	405.22595	-14.68	low score: (3aR,3a1S,5S,5aS,10bS)-methyl 6-acetyl-3	[M+Na]+	Aspidospermatan-type alkaloids	1.546	27.20	55.67	6,018,174.69	141,202.66
3256	9.028	451.19919	-11.50	NCGC00384888-01_C21H32O9_	[M+Na]+	Terpene lactones	1.549	299.35	229.29	5,964,731.14	25,992,626.12
2287	5.638	353.19406	-11.50	low score: vincarine	[M+H]+	Corynanthean-type alkaloids	1.674	55.44	431.17	5,881,042.35	837,439.48
1928	4.601	317.15753	7.79	low score: cinchonine	[M+Na]+	Cinchona alkaloids	1.442	44.00	175.33	5,859,503.37	835,814.99
2929	13.503	415.30447	-17.99	low score: NCGC00385749-01!5-nonadecylbenzene-1,	[M+K]+	Resorcinols	1.324	54.69	7,513.67	5,523,610.32	1,257,974.54
997	4.880	217.14330	0.60	low score: (2R)-2-[(2R,5S)-5-[(2S)-2-hydroxybutyl]ox	[M+H]+	Tetrahydrofurans	1.316	13.75	304.01	5,297,468.28	1,909,478.63
2053	5.294	329.15796	0.46	low score: Propentofylline	[M+Na]+	Xanthines	1.616	57.89	226.48	5,195,604.60	598,544.88
2734	5.976	397.18497	-12.54	low score: 14,15beta-Dihydroxyklaineanone	[M+H]+	Quassinoids	1.578	34.76	351.32	5,147,114.62	855,203.28
2784	5.245	401.21481	-19.24	low score: Reserpic acid	[M+H]+	Corynanthean-type alkaloids	1.499	44.53	682.07	5,121,870.17	11,303,279.20
3483	5.539	475.24988	-3.98	low score: NCGC00169113-03!2-[(2R,4aS,8S,8aS)-8-[	[M-2H2O+H]	Eudesmane, isoeudesmane or cycloeudesmane ses	1.421	53.88	594.46	5,066,885.02	5,299,480.01
1297	4.554	251.12694	-7.73	low score: 5-METHYLCYTOSINE	[2M+H]+	Hydroxypyrimidines	1.530	72.44	4,730.23	4,956,160.56	1,175,823.69
2945	5.899	417.21069	-3.07	low score: Spironolactone	[M+H]+	Spironolactones and derivatives	1.581	54.56	616.48	4,946,341.42	2,589,551.96
2289	5.797	353.19443	-12.54	low score: vincarine	[M+H]+	Corynanthean-type alkaloids	1.631	90.41	978.54	4,919,179.93	862,386.17
2052	5.518	329.15793	0.55	low score: Propentofylline	[M+Na]+	Xanthines	1.409	94.02	987.56	4,902,573.71	1,486,360.62
2785	5.718	401.21487	-19.39	low score: Reserpic acid	[M+H]+	Corynanthean-type alkaloids	1.400	73.74	739.43	4,798,291.22	4,571,579.49
2144	4.378	339.14154	-4.51	low score: Columbamine	[M+H]+	Protoberberine alkaloids and derivatives	1.395	13.59	57.11	4,672,290.93	1,270,390.59
4039	9.595	543.31482	9.55	low score: belladonnine	[M+H]+	Naphthalenecarboxylic acids and derivatives	1.455	38.54	279.81	4,576,784.39	1,825,855.56
3811	6.235	515.28235	14.81	low score: potassium (1R,5aR)-5a,5b,8,8,11a-pentam	[M+Na]+	Triterpenoids	1.532	129.96	0.00	4,559,450.82	9,354,775.05
2207	5.072	345.15219	-6.37	low score: gelsemine	[M+Na]+	Gelsemium alkaloids	1.390	39.96	773.38	4,535,124.92	1,810,545.92
3011	9.706	425.25092	0.21	low score: Caperatic acid	[M+Na]+	Tricarboxylic acids and derivatives	1.449	24.10	204.67	4,145,458.84	3,691,701.80
1897	4.747	313.12714	-18.04	low score: Norclozapine	[M+H]+	Dibenzodiazepines	1.302	15.07	140.28	4,033,465.25	1,969,133.37
1910	4.952	315.14224	19.86	low score: Ranitidine	[M+H]+	Aralkylamines	1.341	76.14	0.00	3,975,500.76	18,587,497.41
3590	11.064	487.19373	0.55	low score: (2R,3S,4S,5R,6R)-2-[[(2R,3R,4R,5S)-3,4-di	[M+K]+	Terpene glycosides	1.440	48.20	324.37	3,915,701.67	2,956,384.46
998	5.121	217.14436	-4.28	low score: (2R)-2-[(2R,5S)-5-[(2S)-2-hydroxybutyl]ox	[M+H]+	Tetrahydrofurans	1.434	67.75	165.16	3,843,301.71	1,354,935.84
1131	5.941	233.15399	-1.72	low score: (+)-Costunolide	[M+H]+	Germacranolides and derivatives	1.426	17.00	215.89	3,750,572.90	8,415,597.32
2050	4.303	329.15707	3.16	low score: Propentofylline	[M+Na]+	Xanthines	1.459	16.41	0.00	3,743,559.94	1,402,950.23
1384	0.660	261.13184	-7.05	low score: 2-(4-oxoquinazolin-3(4H)-yl)ethyl isobutyra	[M+H]+	Quinazolines	1.692	86.94	5,228.29	3,595,295.27	3,321,022.17
3255	9.716	451.19440	-0.89	low score: NCGC00384888-01_C21H32O9_	[M+Na]+	Terpene lactones	1.405	135.02	1,086.68	3,495,793.93	8,018,690.86
3125	4.508	437.23676	-2.45	low score: Nonaethylene glycol	[M+Na]+	Polyethylene glycols	1.702	63.49	230.02	3,264,900.54	17,903,229.15
3824	6.113	517.26196	-3.77	low score: 4-((9S)-3,5,14-trihydroxy-13-methyl-10-((E	[M+Na]+	Cardenolides and derivatives	1.646	24.81	209.68	3,237,314.55	7,466,715.58
2865	7.834	409.18466	-11.41	2-(5-methoxy-4,8,8-trimethyl-2-oxo-2,8,9,10-tetrahyd	[M+H]+	Angular pyranocoumarins	1.800	67.85	369.40	3,094,969.72	11,951,415.65
2870	9.957	409.25793	-3.35	low score: Tricaproin (C6:0)	[M+Na]+	Triacylglycerols	1.560	46.54	377.60	3,042,143.51	2,816,232.74
2188	4.747	343.13742	7.55	low score: Abietin	[M+H]+	Phenolic glycosides	1.325	16.17	950.95	3,011,211.52	2,665,866.80
478	3.540	146.06026	-1.78	6-Quinolinol	[M+H]+	Hydroxyquinolines	1.786	41.25	6,661.62	2,991,907.09	914,008.45
2692	4.175	393.21075	-1.93	Glabrol	[M+H]+	8-prenylated flavanones	1.718	36.22	157.24	2,932,761.22	23,227,424.05
4040	8.978	543.31500	9.22	low score: belladonnine	[M+H]+	Naphthalenecarboxylic acids and derivatives	1.532	46.64	682.04	2,896,051.21	1,186,602.93
2496	4.083	373.14709	7.77	Eleutheroside B	[M+H]+	Phenolic glycosides	1.687	14.74	0.00	2,836,685.27	1,202,486.81
2049	5.697	329.15686	3.80	low score: Propentofylline	[M+Na]+	Xanthines	1.479	54.78	7.47	2,829,777.46	1,917,550.12
1130	10.377	233.15376	-0.73	low score: (+)-Costunolide	[M+H]+	Germacranolides and derivatives	1.316	83.20	605.83	2,648,723.62	6,140,542.34
2206	4.253	345.15167	-4.87	low score: gelsemine	[M+Na]+	Gelsemium alkaloids	1.551	38.18	1,362.91	2,566,152.32	10,549,357.12
2863	8.160	409.18439	-10.75	2-(5-methoxy-4,8,8-trimethyl-2-oxo-2,8,9,10-tetrahyd	[M+H]+	Angular pyranocoumarins	1.731	63.25	211.79	2,432,998.16	11,495,733.84
5793	13.870	869.58313	-11.30	low score: Tilmicosin	[M+H]+	Aminoglycosides	1.397	1,998.71	1,049.91	2,399,182.32	7,706,682.84
3282	4.857	453.21063	-1.41	low score: NCGC00384740-01_C21H34O9_2(1H)-Azul	[M+Na]+	Terpene glycosides	1.577	37.20	3,459.19	2,044,169.63	2,171,757.21
3546	4.771	481.26270	-1.66	low score: Decaethylene glycol	[M+Na]+	Polyethylene glycols	1.736	150.67	683.05	2,023,948.74	13,463,231.92
2636	6.027	387.16348	16.87	Phorbol	[M+Na]+	Tigliane and ingenane diterpenoids	1.575	12.40	231.26	1,965,047.88	1,480,073.71
1804	0.647	305.15784	-2.42	low score: Hexaethylene glycol	[M+Na]+	Polyethylene glycols	1.727	47.36	557.87	1,930,591.71	2,570,746.86
2211	6.566	345.18884	19.29	low score: SB 205607	[M+H]+	Phenylquinolines	1.456	26.77	914.57	1,926,361.95	1,583,142.78
3202	9.771	445.18503	-4.54	low score: NCGC00381245-01_C22H30O8_2-[6-{[(2Z)	[M+Na]+	Elemane sesquiterpenoids	1.445	30.11	360.20	1,832,418.46	1,598,845.27
1894	6.322	313.12598	-14.34	low score: Norclozapine	[M+H]+	Dibenzodiazepines	1.459	35.94	58.12	1,792,786.82	1,436,229.32
2009	7.147	325.23053	-9.47	Denatonium	[M]+	Alpha amino acid amides	1.684	213.70	239.86	1,763,632.46	32,432,571.50
3898	5.010	525.28955	12.32	low score: NCGC00384869-01!4,4,8,10,14-pentameth	[M+K]+	Triterpenoids	1.594	89.34	8.14	1,471,858.40	10,136,224.91
1134	7.615	233.16708	-9.82	low score: Norfentanyl	[M+H]+	Anilides	1.376	487.79	5,211.18	400,103.74	21,240,617.05
