# Supplementary material for: TLC-Derived High-Polar Fractions of Celastrus paniculatus Seeds Attenuate Astrocyte-Driven Microglial Activation Through Suppression of CD40/iNOS Signaling and Pro-Inflammatory Cytokines
Source: Int J Mol Sci. 2026 Apr 16;27(8):3551. doi: 10.3390/ijms27083551 (PMC13116680; doi:10.3390/ijms27083551)
Supplement: Supplementary file 1 [file ijms-27-03551-s001.zip › TOFMassCalibration-1700mzRange_Negative_20251223_093241.pdf]

Tune Report

Instrument Information

**MS Model**	G6545XT	**Run Date/Time**	2025-12-23 09:32:41+0-700
**Serial Number**	SG2219M101	**Last Tuned by**	LCMS
**Firmware Revision**	21.847	**Last Modified by**	LCMS
**Source Type**	Dual AJS ESI	**Slicer Mode**	High Resolution (Position: 5)
**Mass Range**	Low (1700 m/z)	**Instrument Mode**	High Resolution (4GHz)
**Ion Polarity**	Negative	**Tune Type**	TOFMassCalibration-1700mzRange
**SureMass Optimization**	Not Enabled		

Tune File Name TOFMassCalibration-1700mzRange.tun

Negative Polarity Results

TOF Results

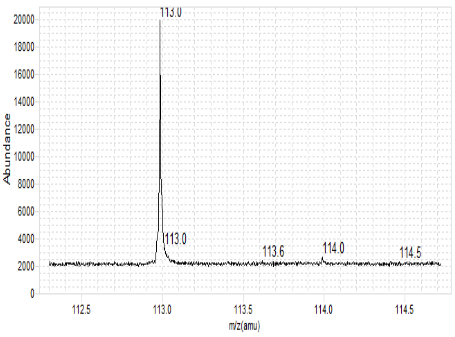

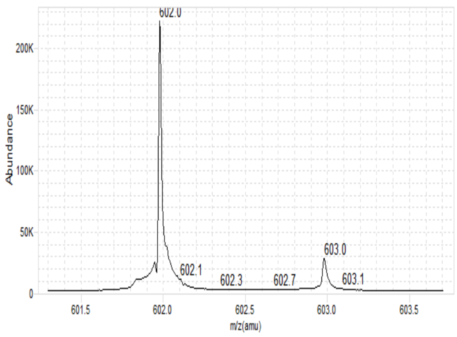

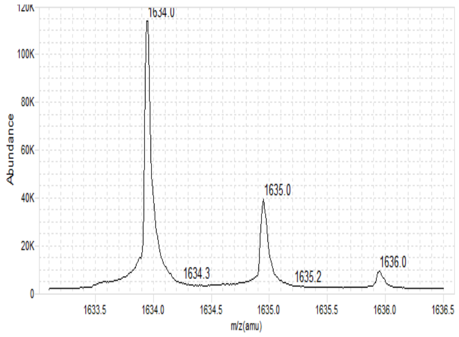

TOF Mass Calibration Data

Theoretical	Actual	Time	Abundance	Calibration Abundance	Resolution	Primary Residuals	Corrected Residuals
112.985587	112.985569	31.743631	18,121	18,291	15,622	0.00	-0.16
301.998139	301.998228	51.257031	32,053	32,843	20,316	0.38	0.30
601.978977	601.978784	71.951672	218,405	221,166	31,651	-0.53	-0.32
1033.988109	1033.988426	93.985870	66,000	66,903	24,707	0.27	0.31
1333.968947	1333.968673	106.615340	109,568	111,023	27,507	0.02	-0.21
1633.949786	1633.949865	117.888001	108,796	110,201	27,379	-0.06	0.05

Setpoints

Source Settings

**Gas Temperature(°C)**	325	**Drying Gas(l/min)**	5.0
**Nebulizer Pressures(psi)**	20	**Capillary(V)**	3500
**Sheath Gas Temperature(°C)**	275	**Sheath Gas Flow(l/min)**	12.0
**Nozzle Voltage(V)**	2000		

Source Actuals

**Gas Temperature(°C)**	325	**Drying Gas(l/min)**	5.0
**Nebulizer Pressures(psi)**	20	**Capillary(V)**	3500
**Cap Current(µA)**	0.090	**Chamber Current(µA)**	0.45

Optics 1

**Fragmentor(V)**	-175	**Skimmer(V)**	-45.0
**Oct 1 RF Vpp(V)**	750	**Oct 1 DC(V)**	-24.9
**Lens 1(V)**	-23.6	**Lens 2(V)**	-15.9
**Lens 2 RF Enable(False/True)**	1	**Lens 2 RF Voltage(V)**	0
**Lens 2 RF Phase(deg)**	180		

Quad

**TTI Quad AMU(amu)**	102.4	**Quad DC(V)**	-22.2
**Post Filter DC(V)**	-22.1		

Cell

**Collision Cell Gas Flow(psi)**	21.0	**Hex RF(V)**	550
**Hex DC(V)**	-19.9	**Hex Delta(V)**	7.0
**Cell Entrance(V)**	-20.4	**Hex 2 RF(V)**	600
**Hex 2 DC(V)**	-12.9	**Hex 2 DV(V)**	1.0

Optics 2

**Ion Focus(V)**	-10.5	**Extractor DC(V)**	9.9
**Lens 3(V)**	59.2	**Bottom Slit(V)**	40.60
**Top Slit(V)**	40.45	**Hex 3 DC(V)**	-11.7

TOF

**Pusher(V)**	-1055	**Pusher Offset(mV)**	-12
**Puller(V)**	700	**Puller Offset(V)**	-32
**Acc Focus(V)**	1960	**Front Mirror(V)**	7000
**Mid Mirror(V)**	1715.4	**Back Mirror(V)**	-1181
**Minimum Mass(m/z)**	50	**Maximum Mass(m/z)**	1700
**Acquisition Rate**	1.0	**Acquisition Time**	1000
**Acq Hold Off Delay (ns)(nsec)**	15000		

Detector

**MCP(V)**	687	**PreAmpOffset(DAC)**	30162
**Low Gain PreAmpOffset(DAC)**	33138	**Gain Abund Ratio**	12.0
**Gain T0 Offset**	-0.088		

Vacuum And Temperatures

**Quad Temp(°C)**	100	**Rough Vac(Torr)**	1.86E+00
**Quad Vac(Torr)**	9.99E+02	**TOF Vac(Torr)**	8.96E-08
**Turbo 1 Speed(%)**	100.0	**Turbo 1 Power(W)**	199
**Turbo 2 Speed(%)**	99.8	**Turbo 2 Power(W)**	27

TOF Mass Calibration Coefficients

**a**	3458.459E-04	**t0**	1008.935E-03	**a2**	2645.710E-15
**b2**	-9651.802E-17	**c2**	1116.026E-18	**d2**	-4141.591E-21
**e2**	0000.000E+00	**f2**	0000.000E+00	**Term Flag**	0x01E0
**Trad**	0	**Poly**	6		

Agilent Technologies
