# Supplementary material for: TLC-Derived High-Polar Fractions of Celastrus paniculatus Seeds Attenuate Astrocyte-Driven Microglial Activation Through Suppression of CD40/iNOS Signaling and Pro-Inflammatory Cytokines
Source: Int J Mol Sci. 2026 Apr 16;27(8):3551. doi: 10.3390/ijms27083551 (PMC13116680; doi:10.3390/ijms27083551)
Supplement: Supplementary file 1 [file ijms-27-03551-s001.zip › TOFMassCalibration-1700mzRange_Positive_20251220_161041.pdf]

Tune Report

Instrument Information

**MS Model**	G6545XT	**Run Date/Time**	2025-12-20 16:10:41+0-700
**Serial Number**	SG2219M101	**Last Tuned by**	LCMS
**Firmware Revision**	21.847	**Last Modified by**	LCMS
**Source Type**	Dual AJS ESI	**Slicer Mode**	High Resolution (Position: 5)
**Mass Range**	Low (1700 m/z)	**Instrument Mode**	High Resolution (4GHz)
**Ion Polarity**	Positive	**Tune Type**	TOFMassCalibration-1700mzRange
**SureMass Optimization**	Not Enabled		

Tune File Name TOFMassCalibration-1700mzRange.tun

Positive Polarity Results

TOF Results

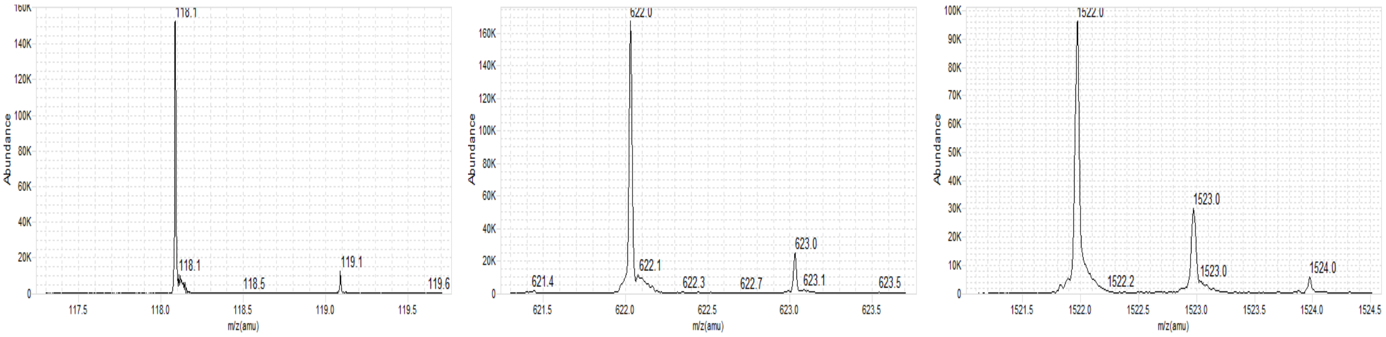

TOF Mass Calibration Data

Theoretical	Actual	Time	Abundance	Calibration Abundance	Resolution	Primary Residuals	Corrected Residuals
118.086255	118.086255	32.424416	164,094	161,108	14,916	-0.13	0.00
322.048121	322.048124	52.890733	220,541	214,754	24,639	0.11	0.01
622.028960	622.028951	73.113790	173,821	170,696	31,888	0.27	-0.01
922.009798	922.009810	88.795699	188,333	190,928	35,285	0.21	0.01
1221.990637	1221.990630	102.072916	145,965	143,986	37,728	-0.09	-0.01
1521.971475	1521.971477	113.797877	94,709	94,920	37,553	-0.37	0.00

Setpoints

Source Settings

**Gas Temperature(°C)**	325	**Drying Gas(l/min)**	5.0
**Nebulizer Pressures(psi)**	20	**Capillary(V)**	4000
**Sheath Gas Temperature(°C)**	275	**Sheath Gas Flow(l/min)**	12.0
**Nozzle Voltage(V)**	2000		

Source Actuals

**Gas Temperature(°C)**	325	**Drying Gas(l/min)**	5.0
**Nebulizer Pressures(psi)**	20	**Capillary(V)**	3999
**Cap Current(µA)**	0.095	**Chamber Current(µA)**	0.47

Optics 1

**Fragmentor(V)**	175	**Skimmer(V)**	45.0
**Oct 1 RF Vpp(V)**	750	**Oct 1 DC(V)**	26.6
**Lens 1(V)**	24.9	**Lens 2(V)**	16.1
**Lens 2 RF Enable(False/True)**	1	**Lens 2 RF Voltage(V)**	0
**Lens 2 RF Phase(deg)**	54		

Quad

**TTI Quad AMU(amu)**	110.2	**Quad DC(V)**	22.9
**Post Filter DC(V)**	22.8		

Cell

**Collision Cell Gas Flow(psi)**	21.0	**Hex RF(V)**	550
**Hex DC(V)**	20.7	**Hex Delta(V)**	-7.0
**Cell Entrance(V)**	21.8	**Hex 2 RF(V)**	600
**Hex 2 DC(V)**	13.7	**Hex 2 DV(V)**	-1.0

Optics 2

**Ion Focus(V)**	9.6	**Extractor DC(V)**	-2.2
**Lens 3(V)**	-64.2	**Bottom Slit(V)**	-42.35
**Top Slit(V)**	-40.80	**Hex 3 DC(V)**	12.5

TOF

**Pusher(V)**	1055	**Pusher Offset(mV)**	-144
**Puller(V)**	-700	**Puller Offset(V)**	28
**Acc Focus(V)**	-1980	**Front Mirror(V)**	-7000
**Mid Mirror(V)**	-1716.8	**Back Mirror(V)**	1181
**Minimum Mass(m/z)**	50	**Maximum Mass(m/z)**	1700
**Acquisition Rate**	1.0	**Acquisition Time**	1000
**Acq Hold Off Delay (ns)(nsec)**	15000		

Detector

**MCP(V)**	850	**PreAmpOffset(DAC)**	30218
**Low Gain PreAmpOffset(DAC)**	33114	**Gain Abund Ratio**	12.0
**Gain T0 Offset**	-0.088		

Vacuum And Temperatures

**Quad Temp(°C)**	100	**Rough Vac(Torr)**	1.84E+00
**Quad Vac(Torr)**	9.99E+02	**TOF Vac(Torr)**	9.32E-08
**Turbo 1 Speed(%)**	100.0	**Turbo 1 Power(W)**	198
**Turbo 2 Speed(%)**	99.8	**Turbo 2 Power(W)**	27

TOF Mass Calibration Coefficients

**a**	3458.830E-04	**t0**	1007.002E-03	**a2**	-1647.924E-12
**b2**	3846.627E-14	**c2**	-6648.524E-22	**d2**	4411.075E-24
**e2**	0000.000E+00	**f2**	0000.000E+00	**Term Flag**	0x0318
**Trad**	1	**Poly**	6		

Agilent Technologies
